# Induced Cognitive Impairments Reversed by Grafts of Neural Precursors: A Longitudinal Study in a Macaque Model of Parkinson's Disease

**DOI:** 10.1002/advs.202103827

**Published:** 2022-02-09

**Authors:** Florence Wianny, Kwamivi Dzahini, Karim Fifel, Charles Robert Eden Wilson, Agnieszka Bernat, Virginie Dolmazon, Pierre Misery, Camille Lamy, Pascale Giroud, Howard Michael Cooper, Kenneth Knoblauch, Emmanuel Procyk, Henry Kennedy, Pierre Savatier, Colette Dehay, Julien Vezoli

**Affiliations:** ^1^ Univ Lyon, Université Claude Bernard Lyon 1 Inserm U1208 Stem Cell and Brain Research Institute Bron 69500 France; ^2^ Primastem Bron 69500 France; ^3^ National Centre for Optics Vision and Eye Care Faculty of Health and Social Sciences University College of Southeast Norway Kongsberg N‐3603 Norway; ^4^ Institute of Neuroscience State Key Laboratory of Neuroscience Chinese Academy of Sciences (CAS) Key Laboratory of Primate Neurobiology Shanghai 200031 China; ^5^ Ernst Strüngmann Institute (ESI) for Neuroscience in Cooperation with Max Planck Society Frankfurt 60528 Germany; ^6^ Present address: International Institute for Integrative Sleep Medicine (WPI‐IIIS) University of Tsukuba Tsukuba Ibaraki 305‐8575 Japan; ^7^ Present address: Laboratory of Molecular Diagnostics Department of Biotechnology Inter‐collegiate Faculty of Biotechnology University of Gdańsk and Medical University of Gdańsk Gdańsk 80‐307 Poland; ^8^ Present address: Laboratory of Experimental Embryology Institute of Genetics and Animal Biotechnology Polish Academy of Sciences Warsaw 05‐552 Poland

**Keywords:** circadian rhythms, cognition and motor control, embryonic stem cells, neural precursors, nonhuman primate low‐dose MPTP

## Abstract

Parkinson's disease (PD) evolves over an extended and variable period in humans; years prior to the onset of classical motor symptoms, sleep and biological rhythm disorders develop, significantly impacting the quality‐of‐life of patients. Circadian‐rhythm disorders are accompanied by mild cognitive deficits that progressively worsen with disease progression and can constitute a severe burden for patients at later stages. The gold‐standard 6‐methyl‐1‐methyl‐4‐phenyl‐1,2,3,6‐tetrahydropyridin (MPTP) macaque model of PD recapitulates the progression of motor and nonmotor symptoms over contracted periods of time. Here, this multidisciplinary/multiparametric study follows, in five animals, the steady progression of motor and nonmotor symptoms and describes their reversal following grafts of neural precursors in diverse functional domains of the basal ganglia. Results show unprecedented recovery from cognitive symptoms in addition to a strong clinical motor recuperation. Both motor and cognitive recovery and partial circadian rhythm recovery correlate with the degree of graft integration, and in a subset of animals, with in vivo levels of striatal dopaminergic innervation and function. The present study provides empirical evidence that integration of neural precursors following transplantation efficiently restores function at multiple levels in parkinsonian nonhuman primates and, given interindividuality of disease progression and recovery, underlines the importance of longitudinal multidisciplinary assessments in view of clinical translation.

## Graphical abstract

This paper shows that successful engraftment of neural precursors restore function at multiple levels in parkinsonian monkeys. Recovery of motor symptoms is accompanied by unprecedented recovery of cognitive performances and chronobiological rhythms and by increased in vivo levels of striatal dopaminergic innervation (dopaminergic transporters).

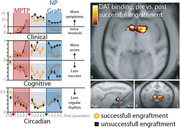

## 1 Introduction

Parkinson's disease is a neurodegenerative condition affecting up to 10M of the worldwide population with incidences increasing with age, making PD the fastest growing neurological disorder in a globally aging population.^[^ ^1^ ^]^ The clinical manifestation is primarily motor with a typical parkinsonian syndrome including bradykinesia, rigidity, and resting tremor and is confirmed by clinical diagnostic tools including in vivo imaging of the denervation of the nigrostriatal dopaminergic (DA) axis.^[^ ^2^ ^]^However, 60–80% of DA cells are lost prior to the onset of clinically diagnosed motor symptoms.^[^ ^3^ ^]^ During this so‐called premotor period preceding the clinical threshold, the DA‐lesion is progressive and accompanied by the manifestation and further deterioration of premotor symptoms including mild cognitive impairments such as early frontoexecutive dysfunctions, ^[^ ^4^, ^5^, ^6^, ^7^, ^8^, ^9^, ^10^, ^11^, ^12^ ^]^ as well as perturbation of circadian rhythm and sleep disorders.^[^ ^13^, ^14^ ^]^ Although considered secondary, premotor symptoms nonetheless have a significant impact on quality of life; the cognitive abilities of the vast majority of PD patients deteriorate leading to psychiatric disturbances,^[^ ^15^ ^]^ with circadian perturbations affecting biological rhythms.^[^ ^16^ ^]^

DA lesion is the hallmark of PD. In addition to circadian regulation, DA lesions impact multiple frontal lobe functions including performance monitoring, motivation, and motricity. There is currently no cure for PD and palliative therapies such as levodopa and deep‐brain stimulation (DBS) mainly correct motor symptoms and often give rise to behavioral perturbations.^[^ ^17^ ^]^ For example, DBS shows no long‐lasting effect on axial symptoms, e.g., freezing of gait and negatively impacts cognitive and neuropsychiatric symptoms.^[^ ^18^, ^19^ ^]^ Importantly palliative therapies do not solve the issue of neuronal loss and the long‐term prognosis. Consequently, cell replacement therapy for PD has been recently reevaluated as a potential cure for PD,^[^ ^20^ ^]^ leading to recent trials aimed at improved grafting procedures, ^[^ ^21^ ^]^ and to pave the way for stem‐cell based transplantation in humans. In this respect, there is a recognized need for more detailed perspectives from preclinical investigations in animal models.^[^ ^22^ ^]^

With the aim of optimizing the therapeutic use of cell replacement in PD, we have examined the global consequence of neural precursor (NP) grafts at different stages of the disease in a macaque model of PD. Numerous efforts have been deployed to use nonmotor cognitive and circadian symptoms as premotor markers for the prognosis of PD.^[^ ^23^ ^]^ However, the multiparametric impact of cell grafts at either preclinical or clinical stages have been insufficiently appraised,^[^ ^17^, ^24^ ^]^ and virtually no studies have addressed the impact of cell replacement on nonmotor cognitive and circadian symptoms.^[^ ^25^ ^]^ This is problematic because early stage PD environment is more favorable for grafted cell survival compared to advanced PD.^[^ ^26^, ^27^ ^]^ Hence in the present study we use an experimental design that allows to address the capacity of cell grafts to reverse the disease at early stages of the motor phase and assess the impact of DA lesion extent on graft efficiency. Importantly, this design also allowed characterizing the dynamics of graft induced recovery by comparing it to spontaneous recovery in the premotor stage. Because nonmotor cognitive and circadian symptoms have a gradual onset prior to clinical manifestations of DA denervation, evaluation of the effects of NP grafts in parkinsonian nonhuman primates (NHP) requires a longitudinal multiparametric investigation, which we have implemented in the present study. We grafted NPs because they hold the promise of enhanced efficiency compared to DA neurons,^[^ ^28^, ^29^, ^30^ ^]^ as in addition to their neurorestorative potential NPs have the capacity to differentiate into neuroprotective astroglial cells.^[^ ^31^, ^32^, ^33^ ^]^

Repeated systemic injections of low‐doses of 6‐methyl‐1‐methyl‐4‐phenyl‐1,2,3,6‐tetrahydropyridin (MPTP), 0.2 mg kg^−1^ every 3–4 days) induced a parkinsonian syndrome with a slow and typically dorsoventral progression of the nigrostriatal denervation, together with a premotor expression of the nonmotor cognitive and circadian symptoms.^[^ ^34^, ^35^, ^36^ ^]^ We implemented a multidisciplinary/multiparametric approach to assess the therapeutic potential of bilateral grafts of NPs in multiple functional domains of the basal ganglia of NHP (*macaca fascicularis*). While our experimental design used a limited number of animals it nevertheless permitted robust statistical analysis thus complying with ethical guidelines, requiring for the minimal number of animals needed to reach sufficient statistical power. In order to adequately demonstrate experimental effects, individual macaques were monitored so that each case constitutes its own control (*n* = 6) and we focused our statistical analyses on *within‐subject control*, which is well established across the field and supported by ethical committees. This required continuous assessment of clinical motor and nonmotor cognitive and circadian symptoms as well as in vivo monitoring of the nigrostriatal lesion with positron emission topography (PET) imaging of DA transporters (DAT) in a subset of animals (*n* = 4). Monitoring was prior to and following the induction of stable motor symptoms,^[^ ^36^ ^]^ and subsequent to transplantation of NPs. In addition, fluoro‐DOPA (^18^F‐DOPA) imaging allowed assessment of the impact of the NP grafts on DA function.^[^ ^37^ ^]^ Finally, postmortem immunohistological examination of NP grafts was performed in order to evaluate the impact of the graft on host tissue as well the survival, integration and differentiation fate of the grafted cells into the host brain.

## 2 Results

**Figure 1:**
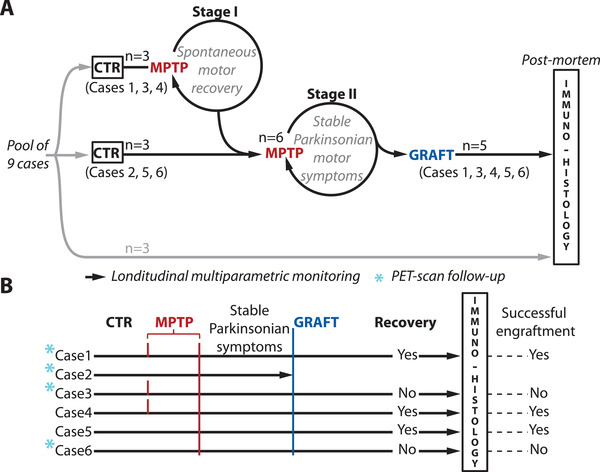
Study design A) Six cases out of nine underwent longitudinal multiparametric monitoring (black arrows), from the control period (CTR) until postmortem evaluation, the three remaining cases were used as immuno‐histological controls of the induced DA lesion. At early stages of MPTP treatment (Stage I, *n* = 3) there is spontaneous recovery from motor symptoms but not from cognitive and circadian symptoms. Continued MPTP intoxication leads to Stage II (*n* = 6) where motor symptoms become permanent. All cases received NP grafts and graft survival was assessed in five cases (case 2 was excluded because of per‐operative brain hemorrhage following graft). B) In all cases (*n* = 6) clinical motor score and nonmotor cognitive and circadian symptoms were monitored. Functional markers of nigrostriatal DA innervation and striatal DA activity were monitored by PET‐scan in a subset of animals (*n* = 4, blue stars). The degree of graft‐integration, evaluated from postmortem immunohistology (successful vs unsuccessful engraftment), conditioned postgraft recovery of motor, nonmotor, and functional markers.

The experimental design (**Figure** **1**; Table S1, Supporting Information) allowed full characterization of MPTP intoxication in 6 animals. We characterized the level of MPTP intoxication in two distinct stages in order to compare spontaneous and graft‐induced recovery. Three animals first received a lesion that brought them to Stage I where they exhibit nonmotor cognitive and circadian symptoms as well as transient motor symptoms^[^ ^34^, ^35^ ^]^ and a nigrostriatal DA denervation superior to 70%.^[^ ^36^ ^]^ In Stage I suspension of MPTP allows spontaneous recovery from motor symptoms to occur and this was investigated in these three individuals (Figure 1A).^[^ ^34^, ^36^, ^38^ ^]^ Monitoring of cognitive, circadian and DA function markers in Stage I makes it possible to characterize spontaneous recovery which can then be compared to graft‐induced recovery. Subsequent to Stage I, monkeys received additional MPTP injections and joined 3 other animals that received de novo MPTP intoxication. In all 6 cases, low‐dose MPTP intoxication proceeded until a clinical score of 10 was reached over at least two consecutive days, subsequently MPTP intoxication was stopped and the clinical score was observed to remain above symptomatic clinical threshold (i.e., motor score > 5, see Section 2.2). This was defined as the Stage II phase, where motor symptoms become permanent.^[^ ^36^ ^]^ This experimental strategy requires establishing for each animal the relationship between cumulated MPTP‐doses and the individual clinical motor scores (Table S2, Supporting Information) thereby taking into account the known individual variability of response to MPTP and allowing adjustment of the intoxication protocol as required (see the Experimental Section). Hence, prior to receiving NP grafts, all animals undergo a prolonged (50–130 days) multiparametric monitoring during which they exhibit a stable expression of symptomatic motor deficits (motor score exceeding clinical threshold, with mild to severe Parkinsonian motor symptoms). Clinical scores and behavioral measures were divided into quantiles (Q1–Q5,^[^ ^34^ ^]^ see the Experimental Section) in order to compare cases on the basis of the presumed DA‐lesion rather than on the time spent in the premotor (MPTP), motor (post‐MPTP/pregraft) and postgraft periods. Following an observation period of 150–270 days subsequent to NP grafts, we performed postmortem evaluation in all longitudinally monitored animals (*n* = 5, case 2 was excluded because of per‐operative brain hemorrhage following graft), and in three additional cases naïve to MPTP intoxication (Figure 1).

### 2.1 Grafted Cells and Postmortem Evaluation

NPs were derived from a rhesus embryonic stem cell line (LYON‐ES1) stably expressing the fluorescent marker tau‐GFP,^[^ ^39^, ^40^ ^]^ which allows precise tracking of the grafted NPs and their integration into the host circuits. RT‐PCR analysis showed that grafted NPs express the typical neural progenitor markers (SOX2, MUSASHI, NESTIN, Figure S1A,D, Supporting Information). Detailed protocols for the derivation of NPs are fully described in the Supporting Information. We chose to graft NPs given their inherent neuroprotective potential resulting from interaction with host cells,^[^ ^41^ ^]^ and their observed higher survival rate compared to mature DA cell grafts.^[^ ^31^, ^42^, ^43^ ^]^ In vitro NPs show both astroglial and neuronal fates and associated marker expression profiles (e.g., GFAP and MAP2 characterizing astroglial and neuronal fates respectively or TH, DRD2, AhD2 distinctive of DA neuronal differentiation, Figure S1, Supporting Information). Hence, we were able to assess the influence of the host environment on the differentiation fate of transplanted cells.

NP grafting was performed 3 months on average after the last MPTP injection (13 ± 6 weeks, see the Experimental Section). We performed bilateral transplantations in multiple functional domains of the basal ganglia in order to potentiate graft efficiency on both motor and nonmotor cognitive and circadian symptoms. The primary target of NP grafts was, in all cases (*n* = 6), the substantia nigra (SN), which provides the source of nigrostriatal DA innervation, i.e., pars compacta, and part of the frontal DA innervation. We additionally targeted striatal regions involved in motor (i.e., posterior putamen—post‐Put) and/or cognitive functions (i.e., anterior caudate nucleus—ant‐CdN). In this manner, we grafted NPs bilaterally in multiple sites including the ant‐CdN, post‐Put and SN with a total of 1.5–4 × 10^6^ NPs per case (see the Experimental Section). Following an average postgraft period of 30 ± 8 weeks, we performed a postmortem immunohistological assessment of tau‐GFP^+^ cells survival and integration into the host milieu (Figure 1).

In two animals, we observed a complete absence of tau‐GFP^+^ cells (Figure S2, Supporting Information). In sharp contrast, we found surviving grafted cells in all the other cases (*n* = 3).

We therefore grouped individual cases according to the outcome of the NPs transplantation, i.e., successful (*n* = 3) or unsuccessful (*n* = 2) engraftment. Cases 1, 4, and 5 were assigned to the *Graft Survival* group and cases 3, 6 to the *No Graft Survival* group.

**Figure 2:**
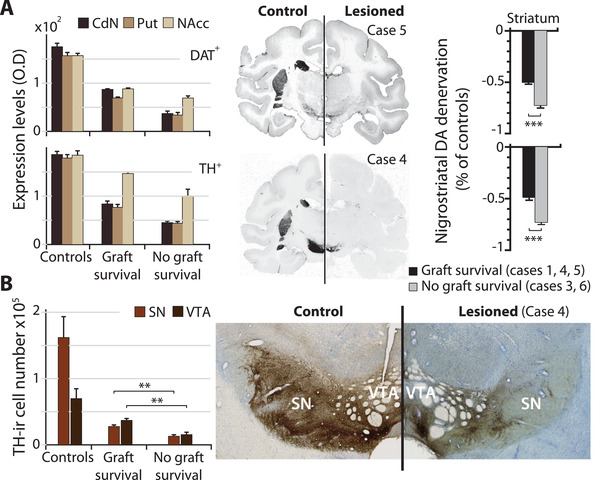
Dopaminergic nigrostriatal lesion. A) Immunostaining for DAT (upper row) and for TH (lower row). Left, optical density (O.D.) measures of DA lesion (arbitrary units) is made on *postmortem* tissue of control animals and following NP grafts on MPTP animals with or without survival of grafted cells. Center, illustrative histological sections showing immunostaining. Right, percent change in expression levels between MPTP and Control animals provides DA denervation estimates by comparing MPTP sub‐groups (Two‐sided Mann–Whitney *U* = 276, *n*1 = 47, *n*2 = 41, ***p < 0.001, *Z* ≥ 5.75). B) Stereological counts of the number TH‐ir cells in SN and VTA (left) and illustrative histological section in corresponding regions. (Two‐sided Mann–Whitney *U* = 0, *n*1 = 2 × 3, *n*2 = 2 × 2, **p ≤ 0.01, *Z* ≥ 2.65, same results for left and right SN or VTA.). Mean ± S.E. *Graft Survival* group: *n* = 3, cases 1, 4, and 5; *No Graft Survival* group: *n* = 2, cases 3 and 6; Control animals group: *n* = 3.

We have previously demonstrated that, in animals expressing stable Parkinsonian motor symptoms following MPTP intoxication, nigrostriatal DA innervation evaluated by striatal DAT binding decreased by more than 80% relative to control measures of striatal DAT levels with a relatively spared binding in the ventral striatum.^[^ ^36^ ^]^ Semi‐quantitative evaluation of postmortem immunostaining for striatal DAT and TH enables us to confirm that the implemented MPTP intoxication protocol (Table S2, Supporting Information, see the Experimental Section) reproduces this dorsoventral pattern of nigrostriatal DA denervation (**Figure** **2**A). We observed a significantly more advanced nigrostriatal denervation in the *No Graft Survival* group (Figure 2A, right).

We further estimated DA lesions by stereological counts of nigral and mesencephalic TH‐positive cells outside the grafts (Figure 2B). The number of TH‐immunoreactive cells (TH‐ir) in the *No Graft Survival* group was reduced by half compared to the *Graft Survival* group (Figure 2B, left). When compared to control animals, i.e., naïve to MPTP intoxication, this resulted in a reduction of TH‐ir cells in the *Graft Survival* group of −83.5 ± 1.4% in SN and −48.2 ± 1.9% in VTA and, in the *No Graft Survival* group, of −92.3 ± 0.6% in SN and −78.9 ± 1.8% in VTA (mean ± SE).

In the group of five animals that underwent postmortem evaluation we observed a binary outcome following NP grafts, i.e., successful (*n* = 3) or unsuccessful (*n* = 2) engraftment and grouped cases accordingly. Given the small numbers of animals per group, a traditional comparison between groups has limited statistical power. Therefore, we used permutation test,^[^ ^44^ ^]^ for the two‐samples of animals to assess the probability of *successful engraftment* over *unsuccessful engraftment*; for this size sample, however, the maximum possible achieved significance level (ASL) is *P* = 0.1 (see the Experimental Section). Note that the multiparametric aspect of the present study considerably increases the power of group comparison. When considering all the parameters we monitored in each animal, all of them displaying the same consistent difference between groups by chance becomes highly unlikely (*P* = 0.031 for 5 variables, under the assumption that each measure is independent—see the Experimental Section).

The longitudinal aspect of the present study increases the statistical power for testing an effect of graft on repeated measures across time. Each case constitutes its own control, so that using linear mixed‐effect modelling we were able to test the *within‐group* longitudinal differences from control (first level contrast, see the Experimental Section). The model considers the difference with respect to the control period (CTR) of the measured variables across conditions as a fixed effect. Random effects were attributed to case and the interaction of case and condition across quantiles. This latter random term accounts for the variance of replications within conditions. Hence, we tested the observed difference with regards to the control period compared to random sample from a population. In the following, we refer to this analysis as the *within‐subject control* difference.

**Figure 3:**
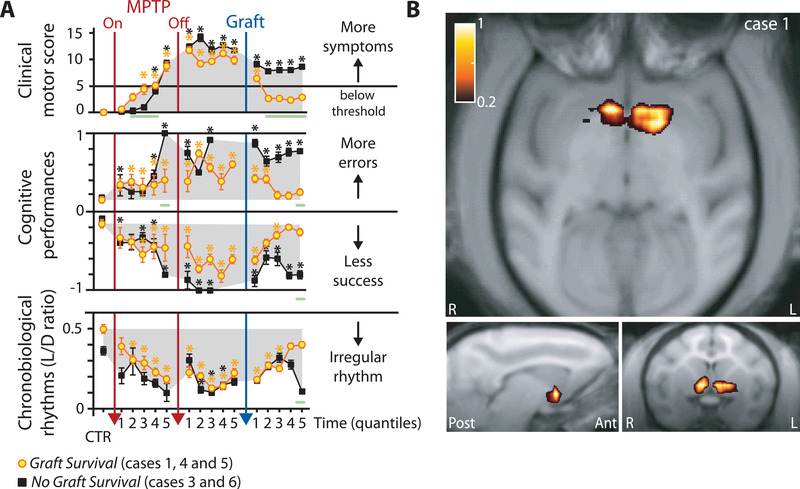
Motor, nonmotor and functional markers of graft‐induced recovery. A) Cases (*n* = 5) are grouped according to the survival (in yellow, cases 1, 4, and 5) or not (in black, cases 3 and 6) of grafted cells evaluated *postmortem*. Motor symptoms clinically scored with the Parkinsonian Monkey Rating Scale (PMRS top panel), cognitive performances (range normalized to control performance, minus one for success, middle panel) and, chronobiological rhythms reflected by the ratio of spontaneous locomotor activity during the light and dark periods (normalized contrast with L/D ratio in control conditions per case, bottom panel). Means ± SE, linear mixed‐effects: * significant *within‐subject control* difference for the *Graft Survival* group (in yellow, *n* = 3—see Table S8 in the Supporting Information) and the *No Graft Survival* group (in black, *n* = 2—see Table S9 in the Supporting Information), two‐sample permutation test (*n* = 5, 120 permutations): green line indicates significant difference between groups *p* ≤ 0.1—see the Experimental Section. Time period per quantile in days: CTR 27 ± 3; MPTP 10 ± 5; post‐MPTP/pregraft 18 ± 8; postgraft 42 ± 12. See Figure S3 (Supporting Information) for weekly view of all individual data points. B) Increase of nigrostriatal innervation in case 1. Parametric maps of DAT binding potential with axial view (top), sagittal view centered on the left hemisphere cluster (bottom‐left panel) and coronal view (bottom‐right panel) of significant positive difference for the contrast pregraft vs postgraft (color scale from 0.2‐1, case 1, *Graft recovery* group). See Figure S4 (Supporting Information) for more details and Figure S10 (Supporting Information) for statistical thresholding in (B) and negative difference.

In order to evaluate differences in behavioral markers due to MPTP intoxication and NP grafts, we tested the *Graft Survival* and the *No Graft Survival* groups separately across all conditions (MPTP, Stage II, and postgraft) and for all conditions across quantiles (**Figure** **3**A and Tables S8 and S9, Supporting Information). Note that all the longitudinal measures (motor, nonmotor and functional markers) were acquired independently of the grouping of animals that was strictly based on postmortem evaluation of graft survival.

### 2.2 Behavioral and Functional Markers of Chronic MPTP‐Induced Parkinsonism

We first verified that MPTP intoxication induced stable motor and nonmotor cognitive and circadian symptoms at Stage II in all cases. During the pregraft period, nonmotor cognitive and circadian symptoms emerged in all animals with progressive deterioration during the premotor period, i.e., before motor scores reached clinical thresholds (Figure 3A; Figure S3 and Tables S8 and S9, Supporting Information).

Alterations of circadian rhythms appeared progressively during the premotor phase (Figure 3A; Figure S3C and Tables S8 and S9, Supporting Information) to reach peak levels during the symptomatic period as previously described.^[^ ^35^ ^]^ This activity pattern of decreased movement during the Light and increased movement during the Dark phases resembles circadian alterations of the sleep–wake cycle observed in PD patients that manifests as increased daytime sleepiness and fragmented sleep structure.^[^ ^45^ ^]^ The premotor and motor alteration of rest‐wake locomotor rhythms was in all cases evidenced by reduced light/dark (LD) ratios (Figure 3A; Tables S8 and S9, Supporting Information). In the *No Graft Survival* group there was high LD ratio variability across cases and significant *within‐subject control* differences could only be observed during the post‐MPTP period (Table S9, Supporting Information). In all cases cognitive performance deteriorated subsequent to and during MPTP‐intoxication (Figure 3A; Figure S3B and Tables S8 and S9, Supporting Information).

During Stage II, both groups displayed a stable parkinsonian syndrome with clinical motor scores ranging from mild to severely symptomatic and significantly impaired cognitive performances (Figure 3A; Figure S3 and Tables S8 and S9, Supporting Information). Circadian rhythms were also reliably reduced throughout Stage II for the *Graft Survival* group (Figure 3A; Table S8, Supporting Information). Note that although MPTP‐lesion induces an attenuation of the amplitude of the locomotor rhythm,^[^ ^34^, ^35^ ^]^ all lesioned animals nevertheless display a strong masking effect of darkness proportionately similar to that of controls which is revealed by the LD ratio.^[^ ^26^ ^]^

### 2.3 Behavioral and Functional Markers Following NP Grafts

Cases with graft survival exhibited robust recovery from motor symptoms with a clinical motor score returning below the symptomatic threshold with no *within‐subject control* difference after the second quantile (Figure 3A; Figure S3A and Table S8, Supporting Information). In contrast, we observed no recovery from motor symptoms in cases with no graft survival (Figure 3A; Figure S3A and Table S9, Supporting Information). Following NP grafts, reduction of the clinical motor score was associated with cognitive and circadian recovery in the *Graft Survival* group. Cognitive performance and circadian rhythms returned to levels observed in the control period on the last 2–3 quantiles postgraft (Figure 3A; Figure S3 and Table S8, Supporting Information). In the *Graft Survival* group, clinical scores progressively and significantly diverged at the 2nd quantile from those of the *No Graft Survival* group, whereas group differences were significant at the last quantile for nonmotor cognitive and circadian symptoms (Figure 3A). Clinical scores dropped below symptomatic thresholds on average 6 weeks following NP grafts, to ultimately achieve maximal motor recovery 5 weeks later (Figures 3A; Figure S1A, Supporting Information). In the *No Graft Survival* group, the initial cognitive and LD ratio recovery shortly after the graft was followed by a relapse on the last three to two quantiles (Figure 3A). Nonparametric circadian rhythm analyses (see the Experimental Section) revealed high variability across cases, preventing postgraft group comparison on those markers (not shown). In case 1 (from the *Graft Survival* group), markers of striatal DA function and DAT increased following NP grafts, in parallel with the observed behavioral recovery (Figure 3; Figure S4B, Supporting Information). Contrasting with the beneficial effects of NP grafts in the *Graft Survival* group, both clinical motor scores and nonmotor cognitive and circadian symptoms persisted following NP graft in the *No Graft Survival* group (Figure 3A; Figure S3, Supporting Information), in agreement with the absence of change in markers of striatal DA function and DAT (Figure S4B, Supporting Information).

In case 1, from the *Graft Survival* group, PET‐scan estimation of nigrostriatal innervation as revealed by DAT binding significantly increased bilaterally in the ventral striatum as early as 15 weeks postgraft (Figure 3B). This suggests a primarily protective influence of grafts on the remaining pool of nigrostriatal projection neurons. Postgraft F‐DOPA scans confirmed a selective effect of NPs on DA synthesis (Figure 3B) that was associated with maximal motor recovery. Significant increases in DA function and DAT binding were also observed in the grafted parts of the striatum of case 1 when compared to the same regions in the *No Graft Survival* group (Figure S4C, Supporting Information).

We performed sham surgery (see the Experimental Section), in two cases monitored for striatal DAT innervation; case 1 from the *Graft Survival* group and case 6 from the *No Graft Survival* group. No recovery from motor nor from nonmotor cognitive and circadian symptoms was observed following sham surgery (Figure S4D, Supporting Information). Further, striatal DAT binding showed no difference between the case with *Graft Survival* versus cases with *No Graft Survival*. There was no specific increase in striatal DAT binding following sham‐surgery (Figure S4E, Supporting Information). Note that ideal sham controls would have required another set of animals following a similar intoxication regimen (leading to stable expression of motor and nonmotor cognitive and circadian symptoms), and sham‐grafted at the exact same locations and to the same extent as the *Graft Survival* group.

### 2.4 Graft‐Induced Recovery Differs from Spontaneous Recovery

**Figure 4:**
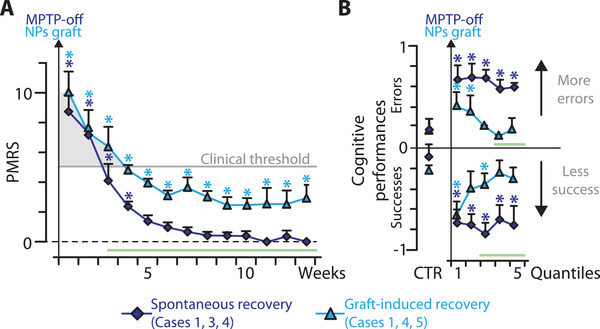
Dynamics of spontaneous versus graft‐induced behavioral recovery. Evolution through time of A) Clinical motor score (over weeks, see Table S10 in the Supporting Information) and B) cognitive performances (over quantiles) for the *Spontaneous recovery* group (in purple, cases 1, 3, and 4) and the *Graft‐induced recovery* group (in blue, cases 1, 4, and 5). Mean ± S.E. across cases, linear mixed‐effects: * significant *within‐subject control* difference for the Spontaneous recovery group (*n* = 3, see Tables S10 and S11 in the Supporting Information) and the graft‐induced recovery group (*n* = 3, see Tables S8 and S10 in the Supporting Information), two‐sample permutation test (*n* = 6, 720 permutations): green line indicates significant difference between groups *p* < 0.05—see the Experimental Section.

We compared the time course of behavioral symptoms and functional markers following graft‐induced recovery to those following spontaneous recovery. In half of the cases (*n* = 3), spontaneous recovery was initially induced by halting MPTP intoxication as described previously (Stage I, Figure 1A).^[^ ^34^, ^36^ ^]^ Spontaneous recovery of clinical motor symptoms displayed faster dynamics and was more complete compared to graft‐induced recovery (**Figure** **4**A). Importantly, spontaneous recovery was not accompanied by changes in cognitive performance which contrasted with graft induced recovery that invariably was accompanied by progressive cognitive improvements (Figure 4B).

These findings are intriguing because spontaneous motor recovery occurs despite an average 71.5 ± 9.7% reduction in striatal DAT relative to control levels and stable symptoms were observed when the lesion reached 82.3 ± 7.9% of control.^[^ ^36^ ^]^

The present findings show that graft‐induced recovery occurs despite an average 78.04 ± 12% reduction in striatal DAT binding (Figure S4C, Supporting Information). These results confirm the specificity of graft‐induced recovery compared to spontaneous recovery, particularly with respect to cognitive deficits (Figure 4B; Tables S10–S11, Supporting Information).

To summarize, we demonstrated that in the *Graft Survival* group there is a recovery from motor and nonmotor cognitive and circadian symptoms to levels that are indistinguishable from the *within‐subject control* period, i.e., before the induced DA lesion. We also showed that the behavioral and functional recovery observed following grafts can be distinguished from the spontaneous recovery observed in Stage I. Furthermore, we show in the *No Graft Survival* group that motor deficits and cognitive impairment persist after grafts at levels that are significantly different from the *within‐subject control* period. Finally, in the subset of animals monitored for striatal DAT levels and DA function, we demonstrate a beneficial effect of NP grafts in case 1 from the *Graft Survival* group whereas no such effect was observed in the two cases from the *No Graft Survival* group. Altogether, these results reveal a congruent difference between groups (*Graft Survival* vs *No Graft Survival*) for all of the five parameters monitored and thus a significant effect of NPs grafts on both behavioral and functional markers (*p* < 0.05).

### 2.5 Behavioral and Functional Recovery Correlate with Graft Survival

**Figure 5:**
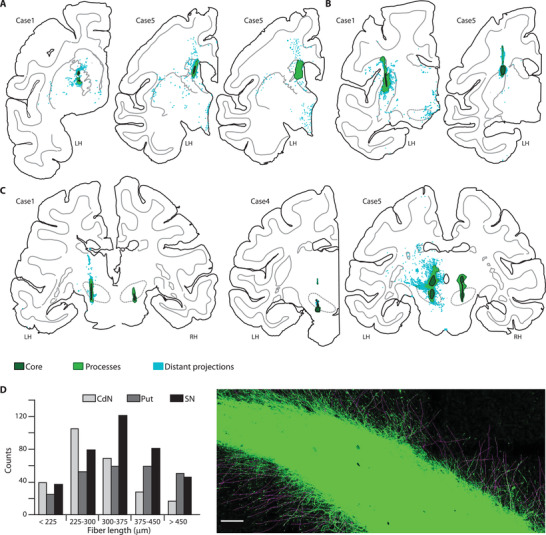
Grafted NPs project over long distances. Digital postmortem reconstructions of NP grafts at the level of the striatum in A) anterior CdN, and B) posterior Put, and C) at the level of the SN (dotted lines). Tau‐GFP+ projection fibers (clear blue) are found at relatively distant locations from the graft core (dark green), much farther than local graft processes (clear green) emanating from the core. See Figure S5 (Supporting Information) for views over the full extent of all grafts. D) Typical processes emanating from the core have an average length superior to 340 µm. Left, count histogram of processes’ length for case 5. Right, typical tau‐GFP+ photomicrograph showing graft‐core and processes at the level of ant‐CdN (case 5, faint purple lines from measurement overlay, scale bar 100 µm).

Surviving grafted cells were identified at different locations including the targeted functional domains of the basal ganglia, i.e., striatum and SN (**Figure** **5**; Figures S5–S9, Supporting Information). The average number of surviving cells was: 1.23 ± 0.9×10^4^ cells in the ant‐CdN, 1.95 ± 1.1×10^4^ cells in the post‐Put, and 5.93 ± 3.0 × 10^4^ cells in the SN (Table S3, Supporting Information) which corresponds to an average survival rate of: 6.9 ± 6.2% in the ant‐CdN, 13.0 ± 7.3% in the post‐Put, and 15.5 ± 9.4% in the SN (overall survival rate of 11.6 ± 4.4% in the *Graft Survival* group, mean ± SE).

We observed unilateral survival of NPs in some of the grafted striatal regions (Table S3, Supporting Information). We could not link asymmetrical survival in the post‐Put to a systematic difference in left versus right motor recovery (Figure S10A, Supporting Information) suggesting the induction of known cross‐hemispheric compensatory processes.^[^ ^46^ ^]^ Nevertheless, small differences were observed in cognitive performance linked to unilateral survival in the ant‐CdN (Figure S10B, Supporting Information); differences that were coupled with a minor unilateral DAT binding decrease in the corresponding striatal region (Figure S10C, right, Supporting Information). In all three cases, the SN was successfully targeted and all surviving grafts displayed extensive tau‐GFP positive projection fibers (Figure 5A–C) which extend several millimeters from the graft core, considerably further than the local processes reaching less than 1 millimeter (Figure 5D). These findings indicate that the *Graft Survival* group is characterized by integration of NPs into the host tissue (Figure 5).

### 2.6 Differentiation Fates Indicate Protective and Restorative Effects of NP Grafts

**Figure 6:**
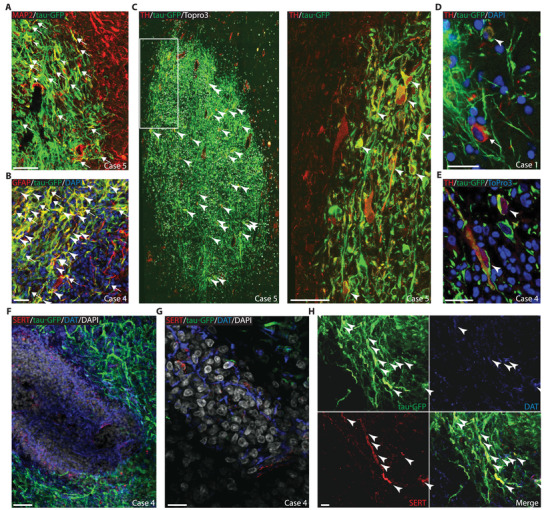
Differentiation Fate of grafted NPs. Illustrative example of NPs differentiated into: A) neuronal cells (arrowheads) and processes (arrows), post‐Put; B) astroglial cells (arrowheads) and processes (arrows), SN; C) aminergic cells, SN (arrowheads, right is enlarged view of region framed on the left); D‐E) aminergic cells (arrowheads) next to host‐aminergic cells (arrow), SN; F‐G) Host tissue within the graft core presenting numerous dopaminergic (DAT^+^, blue) and sparse serotoninergic (SERT^+^, red) processes. H) NPs also display serotoninergic and/or dopaminergic phenotype (arrowheads, case 1). Scale bars, B) 20 µm, C, left) 50 µm, D,E) 100 µm, F) 25 µm, G) 5 µm, H) 15 µm.

**Figure 7:**
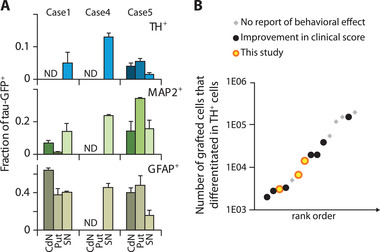
Differentiation Fate of grafted NPs and comparison to literature. A) Fraction of tau‐GFP^+^ colocalized with TH^+^, MAP2^+^, and GFAP^+^. B) Studies reporting quantification of aminergic fate from grafted cells in MPTP monkeys including the present study results (in yellow). *Y*‐axis in log scale, studies referenced in Tables S4 and S5 (Supporting Information).

Detailed immuno‐histological examination of graft locations reveals that grafted NPs differentiated principally into astrocytes and mature neurons (**Figures** **6** and **7**A; Figure S6, Supporting Information). Among surviving NPs, astrocytic fate accounted for 42 ± 5% (GFAP^+^/tau‐GFP^+^ coexpression ratio, Figures 6B and 7A; Table S4, Supporting Information) and neuronal fate for 16 ± 4% (MAP2^+^/tau‐GFP^+^ coexpression ratio, Figures 6A and 7A; Table S4, Supporting Information). Furthermore, we demonstrate that NPs spontaneously differentiate into aminergic (TH+/DAT+) cells thereby potentially restoring a significant proportion of the lost DA pool (Figures 6C–E and 7).

On average, the TH^+^/tau‐GFP^+^ co‐expression ratio implies that 6 ± 2% of tau‐GFP^+^ cells differentiated into aminergic cells (Figures 6C–E and 7A; Table S4, Supporting Information). Up to 13% of tau‐GFP^+^ in SN graft colocalize with TH^+^ expression (case 4), which corresponds to an estimate of 1.43 × 10^4^ TH^+^/tau‐GFP^+^ cells (Figure 7B). Hence, in case 4, after including TH^+^ cells that differentiated from NPs (i.e., TH^+^/tau‐GFP^+^) in the total counts of TH^+^ cells in the SN outside the graft, the DA neuron depletion goes from −86.9% to −78.0% (compared to control nonlesioned animals, Figure 2B). We qualitatively confirmed NP differentiation into both the serotoninergic and the dopaminergic phenotypes (Figure 6H). Together with the protective effect of the graft on the survival or upregulation of DA cells (Figures 3B and 6F,G), these results strongly support a role of the graft in the observed recovery from both motor and cognitive symptoms (Figure 3; Figure S3 and Tables S4 and S5, Supporting Information).

The levels of aminergic differentiation that we report here are well in the range of previous studies reporting an improvement of clinical score following graft (Figure 7; Tables S4 and S5, Supporting Information). Moreover, the astroglial fate is about 7 times higher than the aminergic fate, which could reflect an astroglial‐driven neurorestoration potential of NP grafts.^[^ ^47^, ^48^ ^]^

At some graft sites, aggregates of host cells (i.e., tau‐GFP negative, Figure 6F,G; Figures S8E and S9D,H, Supporting Information) were observed in the graft‐core of nigral transplants, exhibiting aminergic TH^+^, dopaminergic DAT^+^ and sparse serotoninergic SERT^+^ processes (Figure 6F,G; Figures S7D and S8G, Supporting Information). Importantly, no postgraft overgrowth was observed (Figure S11A–C, Supporting Information) and few CD68^+^ macrophages were detected in the vicinity of transplanted sites, despite the absence of immunosuppressive treatment (Figure S11D–F, Supporting Information).

**Figure 8:**
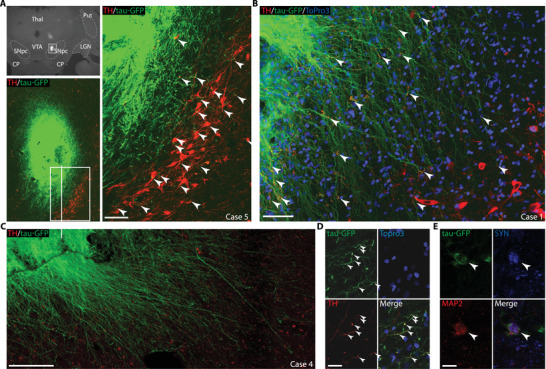
Histological evidence of NP integration and interaction with host cells. A) Photomicrographs (up‐left panel) shows position of graft in SN, framed region zoomed in bottom‐left panel. Right‐panel show zoom of framed region in the bottom‐left panel. In (A) and (B), grafted NPs (tau‐GFP^+^, green) extend processes (arrowheads) toward surviving host DA cells (SN grafts). C) NPs display numerous extensive processes projecting outside the graft‐core. D) NPs differentiate into aminergic phenotype displaying TH^+^ processes (arrowheads, case 1). E) Synaptophysin expression (SYN, blue) co‐localized with grafted cell (tau‐GFP^+^) differentiated into mature neuron (MAP2^+^) suggesting synaptic contact (arrowhead, case 5). Scale bars: A–C) 100 µm, D,E) 5 µm.

Finally, we explored the extent to which the grafted NPs were structurally integrated, via contacts with host neurons. For this purpose, we examined synaptophysin (SYN) immunohistology. SYN is localized in the membrane of presynaptic vesicles and provides a specific and sensitive marker for synapses.^[^ ^49^ ^]^ We explored regions at the periphery of the grafts, where the numerous local processes emanating from the core appear preferentially directed toward host DA cells (**Figure** **8**A,C). Confocal microscopy shows that SYN co‐localized at the junction between TH^+^ and MAP2^+^ host‐cells and tau‐GFP^+^ processes from grafted‐cells (Figure 8E; Figure S9E,G,I–K, Supporting Information), indicating that local processes stemming from the graft core establish synaptic contacts with the remaining host DA cells in the SN (Figure 8). Together with the presence of long‐distance projections emanating from the grafted sites (Figure 5), this suggests functional integration of NPs into the host brain circuitry supporting the role of NP grafts in the observed functional recovery (Figure 3A,B; Figure S3 and S4, Supporting Information).

## 3 Conclusion

We transplanted macaque NPs into multiple functional domains of the basal ganglia, in both hemispheres of middle‐aged parkinsonian macaques. Our study demonstrates that the successful integration of the grafts promotes a robust clinical and unprecedented cognitive recovery (Figure 3A; Figure S3, Supporting Information), as well as partial circadian recovery. Within individuals, i.e., longitudinally, the observed changes on behavioral markers were not randomly distributed across periods. Hence, the observed recovery from clinical, cognitive and circadian impairments following NPs graft was statistically significant (see Table S8 in the Supporting Information). Recovery is accompanied by quantitative effects on functional markers of nigrostriatal DA innervation and striatal DA function in the subset of animals tested (Figure 3B; Figure S4B,C, Supporting Information).

This study thus provides empirical evidence in favor of a promising stem‐cell derived cell therapy for PD patients. First, we show for the first time that transplantation and successful engraftment of NPs efficiently promotes recovery from motor, nonmotor cognitive and circadian symptoms. Second, we demonstrate the restorative and the neuroprotective potential of grafted NPs. Our results show that NPs have the capacity to restore a significant pool of mature neurons (Figure 6A), including dopaminergic and serotoninergic cells. In addition, NPs largely differentiate into astroglial cells that the present study confirms provide a neuroprotective environment for the surviving pool of DA neurons.^[^ ^31^, ^32^, ^33^ ^]^ Third, our results also suggest that nigral transplantation of NPs provide neurotrophic effects on the host cells (Figures 6F,G; Figures S8E,G–J and S9D,H, Supporting Information), orienting endogenous cells toward a DA fate. ^[^ ^50^, ^51^ ^]^ Finally, we provide cellular evidences of structural and functional integration of grafted NPs (Figures 5 and 8; Figure S9E,G,I–K, Supporting Information) into the host brain network.

The consistent cognitive recovery following successful engraftment of NPs in ant‐CdN and SN establishes the potential of cell therapy to alleviate nonmotor cognitive symptoms in addition to motor symptoms in PD patients. The specificity of the graft‐induced cognitive recovery (Figure 3A) was further confirmed by the absence of cognitive improvements following sham‐surgery (Figure S4D, Supporting Information) and spontaneous motor recovery (Figure 4B). These results anticipate a beneficial outcome of cell therapy for the majority of PD patients experiencing mild cognitive impairment (MCI) that significantly affects their quality of life.^[^ ^52^ ^]^ Cognitive functions in human and NHP depend on the integrity of the prefrontal cortex (PFC), the DA system and the DA innervation of PFC via direct projections from SN and ventral tegmental area.^[^ ^53^, ^54^, ^55^, ^56^, ^57^, ^58^ ^]^ In addition, frontostriatal circuits are also extensively involved in cognitive function and more particularly the cognitive sectors of the striatum, i.e., ant‐CdN.^[^ ^59^ ^]^ Many cognitive symptoms expressed in PD patients arise not only from DA degeneration but also from other monoamine neurotransmitter systems closely related to the DA system such as serotonin and norepinephrine.^[^ ^10^, ^60^ ^]^ These systems are also affected in the MPTP‐monkey model.^[^ ^61^ ^]^ The cognitive task that was used in our study, allows directly relating the increased errors and reduced success rates observed in Stages I and II to the reduced control over motor planning that parallels executive dysfunctions observed in PD patients.^[^ ^13^, ^62^ ^]^ Hence, the observed reduction of cognitive performance (Figure 3A; Figure S3B, Supporting Information) can be directly imputed to the nigrostriatal DA lesion, the altered DA innervation of frontal cortex,^[^ ^53^ ^]^ and the wider MPTP‐induced alteration of the aminergic system including noradrenalin and serotonin.^[^ ^38^, ^63^, ^64^, ^65^, ^66^ ^]^ We relate the consistent cognitive recovery following NP grafts (Figure 3A) to the efficiency of the transplanted NPs in both SN and ant‐CdN where an average of, respectively 5.93 ± 3 × 10^4^ and 1.23 ± 0.9 × 10^4^ cells survived, differentiated (Figure 6) and integrated the host network through local and long‐distance projections (Figures 5 and 8). Importantly, we show that grafted NPs can differentiate into a substantial proportion of TH^+^ expressing cells (Figure 7A), well within the range of previous studies reporting clinical improvement (Table S4, Supporting Information), but also in the serotoninergic phenotype (Figure 6H) thereby supporting their role not only in motor but also in cognitive recovery.

In addition to these restorative effects, our results suggest that bilateral multisite NP grafts exert a neuroprotective influence on the pool of surviving DA cells (VTA and medial part of dorsal SN, Figure 2B) and of their ventro‐striatal target.^[^ ^59^, ^67^, ^68^ ^]^ We show a significant and specific increase in striatal DAT binding following graft (Figure 3B) and not following sham‐surgery (Figure S4E). This increase in DAT binding is located in the ventral striatum, which is the target of the surviving pool of mesostriatal DA terminals, i.e., originating from the medial part of the dorsal SN and VTA.^[^ ^67^, ^68^ ^]^ Interestingly, we previously demonstrated that the ventral striatum is preferentially spared in MPTP‐lesioned animals following induced spontaneous recovery.^[^ ^36^ ^]^ The large proportion of astroglial differentiation (Figure 7A) might support this protective effect of NP grafts. Direct brain infusion of GDNF in both parkinsonian monkeys and PD patients have been shown to lead to increased proportion of DA neurons and fibers,^[^ ^69^, ^70^ ^]^ accompanied by improved motor and DA function.^[^ ^69^, ^71^, ^72^ ^]^ GDNF release by astrocytes is known to provide direct trophic and neuroprotective effects on the host DA pathways.^[^ ^73^ ^]^ Importantly, the large number of astroglial cells we observed in the grafts is therefore competent for inducing behavioral recovery through provision of neurotrophic factors such as GDNF,^[^ ^47^, ^48^ ^]^ in accordance with previous reports on human NP grafts in MPTP‐monkeys showing differentiation into astrocytes releasing GDNF.^[^ ^74^ ^]^ However, for striatal GDNF infusion to be efficient nigrostriatal denervation must be at early stages,^[^ ^75^ ^]^ even when combined with SN infusion,^[^ ^76^ ^]^ because the alpha‐synuclein accretion that occurs with disease progression negatively impacts the competence of DA neurons to respond to GDNF.^[^ ^77^ ^]^ We can therefore conclude that the consistent recovery of function that we observe following integration of NP grafts likely results from the astroglial neuroprotective, trophic and regenerative effects combined with the restoration of a significant proportion of DA cells. Although we report some degree of recovery of DAT binding in the grafted caudate nucleus and putamen (Figure S4B,C, Supporting Information), these effects were minimal compared to what was found in the ventral striatum. However, longer postgraft survival could lead to greater increases in DAT binding. Indeed, transplanted PD patients display fast clinical improvements in motor function, i.e., within 2–3 months after transplantation, whereas it can take 1–2 years to detect via functional imaging significant increase in DA function at the grafted locations.^[^ ^78^, ^79^, ^80^, ^81^, ^82^, ^83^ ^]^

The impact of cell therapy on chronobiological rhythms as far as we know has not been previously studied in the MPTP‐monkey model. The initially observed circadian recovery following NP grafts concerned both groups but was followed very quickly by a deterioration after the third quantile in the *No Graft Survival* group. However, in the *Graft Survival* group the LD ratio recovery was significant but only partial (Figure 3A). Previously we showed that following MPTP‐induced nigrostriatal DA lesion, environmental cues mask the failure of the intact SCN to drive striatal clock genes and DA functions that control rest‐wake locomotor rhythms.^[^ ^35^ ^]^ This raises the possibility of complex interactions between circadian rhythms and cognitive recovery, given the role of circadian dysfunction on cognitive impairments.^[^ ^84^ ^]^ Here, the partial recovery of circadian rhythms in the presence of environmental timing cues (Figure 3A), could reflect incomplete restoration of DA function. However, following NP grafts, under continuous light conditions (*n* = 4, cases 1, 3, 4, and 5) there was an absence of endogenous circadian rhythm as observed in the MPTP monkey.^[^ ^35^ ^]^ This again points to NP grafts failing to fully restore the DA network involved in the SCN control of rest‐wake rhythms. DA projection neurons from SN and VTA regulate sleep‐wake rhythms through interaction with several structures from the circadian network including the locus coeruleus, the lateral hypothalamus and the pedunculopontine nucleus.^[^ ^85^ ^]^ These structures, which were not grafted in the present study, are impacted in PD patients and following MPTP intoxication thus constituting future candidates for cell replacement protocols.^[^ ^86^, ^87^, ^88^ ^]^

There are known limitations in the use of cell‐therapy for PD, e.g., presence of Lewy‐body formations in the grafted cells.^[^ ^89^, ^90^ ^]^ However, the benefit‐risk balance of cell‐therapy, even in cases of Lewy‐body development in the grafted cells, would seem clearly in favor of the transplanted patient.^[^ ^20^ ^]^ Three decades after the first clinical trials of transplanting cells from fetal ventral mesencephalon in PD patients,^[^ ^91^ ^]^ cell replacement therapy in PD is still not a proposed treatment. However, this therapeutic approach has recently been shown to be efficient at different levels,^[^ ^82^, ^83^, ^92^ ^]^ leading to more recent trials.^[^ ^93^ ^]^ In parallel, many strategies have been developed in recent years in order to improve the safety and efficiency of using ESCs or iPSCs as a source for the production of the to‐be‐transplanted cells.^[^ ^30^ ^]^ Virtually no NHP studies have addressed the impact of cell replacement on nonmotor circadian symptoms and only one recent reported beneficial effects on depressive symptoms (Table S5, Supporting Information). Here we report for the first‐time efficient recovery from cognitive and clinical motor symptoms, as well as partial circadian recovery following NP grafts in the NHP model of PD. The present study confirms the neuroprotective and restorative capacities of NP grafts and, underlines the crucial importance of longitudinal and multifaceted approaches for a better translation to the clinic and appraisal of early‐phase clinical trials.

While there are doubtless limitations in the use of the MPTP‐monkey model to study human‐specific aspect of PD; this model remains nevertheless the gold standard for modeling PD and for the preclinical evaluation of new therapeutic approaches. For instance, previous studies have shown that advanced nigrostriatal lesion is detrimental to the survival of grafted cells,^[^ ^26^, ^27^ ^]^ which is coherent with our observation of poor survival of grafted NPs in the *No Graft Survival* group of animals that did not recover. Further, in the *No Graft Survival* group immune rejection is likely to have occurred as previously observed following allogenic transplantation in nonimmunosuppressed monkeys.^[^ ^94^, ^95^ ^]^ One conclusion from the present study is that future therapeutic cell replacement procedures should be carried out preferably at early stages of PD.

## 4 Experimental Section

In agreement with the 3Rs,^[^ ^96^ ^]^ the rationale for the current study is that each case represents its own control through detailed follow‐up of all the periods of the protocol, i.e., CTR, MPTP, post‐MPTP/ pregraft, and postgraft. Precise and detailed reports for each clinical, behavioral, and functional parameter followed were published as well as for the characterization of NPs grafted in the present study. These precise and detailed control procedures are described in Table S1 (Supporting Information). All monkeys were closely monitored on a regular basis throughout the day, by researchers and animal care staff, in order to ensure that levels of health and welfare were strictly maintained, particularly during the MPTP period. Adaptations to housing and feeding procedures were made in direct response to individual symptoms in the MPTP phase, for example adaptations of water provision to ensure monkeys were able to drink ad libitum.

### Study Design

The objective of the research was to investigate the efficiency of NP grafts to restore motor and nonmotor cognitive and circadian symptoms in parkinsonian macaque monkeys. All behavioral and functional measures were acquired by experimenters blinded to the outcome of the grafts integration as this was evaluated postmortem. Postmortem quantifications of graft survival, differentiation rate, and length of graft's processes were done by investigators blinded to the precise evolution of parkinsonian symptoms of the animals following graft. The experimental findings included replication of the procedure in all individual cases separately and conclusions on the findings arose from difference in outcome based on postmortem evaluation of grafts integration. Cases followed by PET imaging were chosen randomly before experiment starts as well as cases chosen for sham‐grafts. Data collection was stopped at least 7 months following transplantation and after last possible PET‐scan acquisition. Data‐points of the locomotor activity acquired on the day of anesthesia for MRI, PET‐scan or surgery were excluded from circadian analyses. Presented results included all other data‐points, no outliers were excluded. Nine macaque monkeys (Macaca fascicularis, *n* = 9) were used in total for this study. The study was designed such that one third (*n* = 3) were randomly selected as histological controls, i.e., never received MPTP injections and, the other two third (*n* = 6) entered the MPTP protocol. While control animals were selected for histological comparison, the study was designed such that each animal would be its own control for clinical, behavioral and functional measures. Figure 1A displays a schematic representation of the study design and protocol. Two third of the MPTP group (*n* = 4) were randomly selected to be followed by PET imaging of striatal DA transporters and DA function throughout the full study and, half of them (*n* = 2) were randomly selected to evaluate sham‐grafts effects on clinical, behavioral, and functional measures. In addition, half of the animals entering the MPTP protocol were randomly selected (*n* = 3) for a two‐step MPTP procedure in which MPTP injections were first halted based on clinical score,^[^ ^34^ ^]^ in order to induce spontaneous recovery for further comparison of this *spontaneous* recovery to the prospective *graft‐induced* recovery. MPTP‐intoxication was then resumed in order to induce stable motor symptoms over the period preceding cell therapy.^[^ ^36^ ^]^ Six late middle‐aged—11–13 (13–17) years old at protocol onset (end) female macaque monkeys (Macaca fascicularis, 4–5 kg) were intoxicated with low‐dose 6‐methyl‐1‐methyl‐4‐phenyl‐1,2,3,6‐tetrahydropyridin injections (MPTP, 0.2 mg kg^−1^, i.m.). Animals were housed in a room dedicated to MPTP experiments, with free access to water and received food twice a day. The neurotoxin was delivered at low‐doses chronically each 3–4 days (slowly progressive lesion) during prolonged periods, followed by low‐doses intoxication at higher frequency over 3 weeks (daily injections on weekdays, HF in Table S2 of the Supporting Information) in order to ensure stable persistent motor symptoms, as described previously.^[^ ^36^, ^38^ ^]^ Cases 1, 3, and 4, were selected to critically compare the evolution of clinical, behavioral and functional markers following spontaneous clinical motor recovery,^[^ ^34^, ^36^ ^]^ with those obtained during potential recovery induced by cell therapy. In order to do so, the slowly progressive lesion induced by chronic low‐dose MPTP injections was suspended as soon as the clinical score reached symptomatic threshold (Figure S3 and Table S2, Supporting Information). In all cases, low‐dose MPTP injections induced persistent motor‐symptoms. Daily MPTP injections were cautiously stopped when the PMRS‐motor score was above 10 for two consecutive days following one MPTP injection. For results presentation, animals were grouped according to the outcome of NP grafts (i.e., successful vs unsuccessful engraftment) at the end of the protocol following cell therapy (see Figure 1), i.e., first group—*Graft Survival* (*n* = 3, cases 1, 4, 5) and, second group—*No Graft Survival* (*n* = 2, cases 3 and 6). Case 2 had per‐operative brain hemorrhage following NPs transplantation and was thus excluded from group comparison. Data were presented according to the following periods of the protocol: 1) CTR (measures acquired before MPTP‐onset); 2) MPTP (during MPTP intoxication period); 3) post‐MPTP (following arrest of last MPTP injections, clinical motor score remained stably above 5 during this period, i.e., motor‐symptomatic); and 4) postgrafts (following transplantation). Delays between last MPTP injection and cell therapy were 4–23 weeks (*n* = 6, all cases) and, delays between last MPTP injection and sham‐grafts were 6–16 weeks (*n* = 2, cases 1 and 6).

### Transplanted Cells

NPs were derived from a rhesus embryonic stem cell (RhESC) line stably expressing tau‐GFP (LYON‐ESC line).^[^ ^39^ ^]^ NPs were obtained either as described previously,^[^ ^97^ ^]^ and amplified in the presence of EGF and FGF2 (NPs, Figure S1A, upper panels, Supporting Information) or after MS5 induced‐neural differentiation,^[^ ^98^ ^]^ followed by early midbrain DA differentiation (mDA‐NPs, Figure S1A, lower panels, Supporting Information). RT‐PCR analysis returned similar marker expression profiles (except for the specific DA markers LMX1A and LMX1B which are not expressed in NPs; Figure S1A,B, Supporting Information).

Because of space restriction, the complete details concerning Parkinsonian Monkey Rating Scale—PMRS, surgical procedures, transplanted cells, cognitive behavior—detour task, circadian rhythm follow‐up, dopamine function imaging—[^11^C]‐PE2I and [^18^F]‐FDOPA, immunohistological, and quantification procedures can be found in the Supporting Information.

### Statistical Analysis

Data were segmented for MPTP, post‐MPTP, and postgraft periods into five equal epochs and grouped variables into each of these epochs. This method of segmentation, described previously,^[^ ^34^, ^36^ ^]^ thus reveals normalized stages of the progression of processes and allows for comparison of different parameters across an equivalent number of epochs for all subjects, called quantiles (27 ± 3 days for the CTR period; duration per quantile for premotor 10 ± 5 days, motor/pregraft 18 ± 8 days and postgraft 42 ± 12 days). Results were presented as means ± standard errors. If not stated otherwise, group comparison was made with a permutation test.^[^ ^44^ ^]^ To test how likely it is that all five animals present the same changes of behavioral parameters following NP grafts, a permutation test applied to the two samples of animals was used, i.e., those displaying successful engraftment (*m* = 3) and those displaying unsuccessful engraftment (*n* = 2). The power of Treatment over Control was thus explicitly tested (here treatment refers to successful engraftment and Control to unsuccessful engraftment) under the null hypothesis that both distributions were the same. For *N* = 5, there were 120 unique permutations. However, there were only $\frac{N!}{m!n!}=\frac{5!}{3!2!}\;$ = 10 unique combinations for dividing N individuals into two groups of size *m* and n. Thus, the maximum achieved significance level (ASL) could attain at most a value of *p* = 0.1, with any three of the five animals possibly assigned to the Treatment group.^[^ ^44^ ^]^ Hence, if the permutation test on, e.g., clinical score reaches the maximum ASL, it means that there is a 1 in 10 chance of getting a difference between the two sample means as large as that observed, under the assumption that the two distributions are the same. Now if all the behavioral parameters were considered that followed per animal (*n* = 5), the chance of having all of them displaying the same consistent difference between groups is equal to$\;\frac{1}{2^{n}}$. Thus, there is 1 in 32 chance (*p* = 0.03) of getting the same sign of the difference between the two sample means for all four parameters, under the assumption that each measure is independent. In order to consider each case as its own control, the longitudinal dataset was analyzed using linear mixed‐effects modeling to test the longitudinal difference to first level, i.e., *within‐subject* control period, for all cases as a group. In this way, both fixed‐ and random‐effects terms were incorporated in a linear predictor expression from which the conditional mean of the response can be evaluated, taking into account the longitudinal aspect of the study, i.e., the nonindependence of repeated measures performed per case across the different conditions (CTR, MPTP, post‐MPTP/pregraft, postgraft). The linear mixed‐effect model used considered the difference to CTR of the measured variable across conditions as a fixed effect. Random effects were attributed to case and the interaction of case and condition across quantiles. This latter random term accounted for the variance of replications within conditions (i.e., MPTP, post‐MPTP, and postgraft). Hence, instead of testing *Graft Survival* versus *No Graft Survival* groups, e.g., postgraft, the observed difference was tested per group with regard to *within‐subject control* compared to random sample from a population. A significant *within‐subject control* difference thus indicated that, e.g., MPTP intoxication induced cognitive impairments. Whereas an absence of significant *within‐subject control* difference indicated that, e.g., NPs graft induced a recovery from cognitive impairments, to a level that is not distinguishable from that reached before the DA lesion. Detailed statistics corresponding to significance tests in Figures 3A and 4 are reported in Tables S8–S11 (Supporting Information).

Significance was considered at *p* < 0.05. Statistical analyses were computed using R software (R Foundation for Statistical Computing, Vienna, Austrian http://www.R‐project.org) and the lmer function from lme4 package.^[^ ^99^ ^]^ The summary method for the results from the lmer function fitting the linear mixed‐effects model did not provide *p*‐values because of uncertainties in the correct attribution of degrees of freedom, see ref. [99]. Significance was assessed based on exclusion of 0 in the 95% confidence intervals that were calculated by a profiling method.

### Study Approval

All procedures were carried out according to the 1986 European Community Council Directives (86/609/EEC) which was the official directive at the time of experiments, the French Commission for animal experimentation, the Department of Veterinary Services (DDSV Lyon, France). Authorization for the present study was delivered by the “Préfet de la Région Rhône Alpes” and the “Directeur départemental de la protection des populations” under Permit Number: #A690290402. All procedures were designed with reference to the recommendations of the Weatherall report, “The use of nonhuman primates in research.”

## Conflict of Interest

The authors declare no conflict of interest.

## Author Contributions

H.K, P.S, C.D and J.V are senior authors. Data acquisition: F.W., K.D., K.F., A.B., V.D., and J.V.; Data analysis: F.W., K.D., K.F., P.M., C.L., K.K., and J.V.; Parkinsonian Monkey Rating Scale: K.D., K.F., C.R.E.W., E.P., and J.V.; Cognitive testing: K.D., K.F., E.P., and J.V.; Circadian analysis: K.F., H.M.C., and J.V.; PET‐scan: K.D., C.R.E.W, E.P., and J.V.; PET‐scan analysis: J.V.; Cell culture and NPs production for graft: F.W., A.B., V.D., P.S., and C.D.; Surgical grafts: K.D, P.G, E.P and J.V; Histology: F.W, K.D and J.V; Project proposal: H.K., P.S., and C.D.; Experimental design: H.M.C., E.P., H.K., P.S., C.D., and J.V.; Writing of the first draft: F.W., H.K., J.V. with the assistance of C.R.E.W., P.S., and C.D. All authors edited and commented on the manuscript.

## Supporting information

Supporting Information

## Data Availability

Research data are not shared.
