# Supplementary material for: Induced Cognitive Impairments Reversed by Grafts of Neural Precursors: A Longitudinal Study in a Macaque Model of Parkinson's Disease
Source: Adv Sci (Weinh). 2022 Feb 9;9(10):2103827. doi: 10.1002/advs.202103827 (PMC8981458; doi:10.1002/advs.202103827)
Supplement: Supplementary file 1 — Supporting Information [file ADVS-9-2103827-s001.pdf]

ADVANCED SCIENCE
Open Access

Supporting Information

for *Adv. Sci.*, DOI 10.1002/advs.202103827

Induced Cognitive Impairments Reversed by Grafts of Neural Precursors: A Longitudinal Study in a Macaque Model of Parkinson's Disease

*Florence Wianny, Kwamivi Dzahini, Karim Fifel, Charles Robert Eden Wilson, Agnieszka Bernat, Virginie Dolmazon, Pierre Misery, Camille Lamy, Pascale Giroud, Howard Michael Cooper, Kenneth Knoblauch, Emmanuel Procyk, Henry Kennedy, Pierre Savatier, Colette Dehay and Julien Vezoli**

ADVANCED SCIENCE
Open Access

Supporting Information

for *Adv. Sci.*, DOI: 10.1002/advs.202103827

Induced Cognitive Impairments Reversed by Grafts of Neural Precursors: A Longitudinal Study in a Macaque Model of Parkinson's Disease

Florence Wianny, Kwamivi Dzahini, Karim Fifel, Charles R.E. Wilson, Agnieszka Bernat, Virginie Dolmazon, Pierre Misery, Camille Lamy, Pascale Giroud, Howard M. Cooper, Kenneth Knoblauch, Emmanuel Procyk, Henry Kennedy, Pierre Savatier, Colette Dehay and Julien Vezoli**

Supporting Information

Induced Cognitive Impairments Reversed by Grafts of Neural Precursors: A Longitudinal Study in a Macaque Model of Parkinson's Disease

Florence Wianny[1, 2], Kwamivi Dzahini[1, 2], Karim Fifel[1, †], Charles R.E. Wilson[1], Agnieszka Bernat[1, ‡, §], Virginie Dolmazon[1], Pierre Misery[1], Camille Lamy[1], Pascale Giroud[1], Howard M. Cooper[1], Kenneth Knoblauch[1,3], Emmanuel Procyk[1], Henry Kennedy[1, 4, +], Pierre Savatier[1, 2, +], Colette Dehay[1, 2, +] and Julien Vezoli[1, 5,+,]*

[1] Univ Lyon, Université Claude Bernard Lyon 1, Inserm U1208, Stem Cell and Brain Research Institute; 69500 Bron, France.

[2] Primastem; 69500 Bron, France.

[3] National Centre for Optics, Vision and Eye Care, Faculty of Health and Social Sciences, University College of Southeast Norway, N-3603 Kongsberg, Norway.

[4] Institute of Neuroscience, State Key Laboratory of Neuroscience, Chinese Academy of Sciences (CAS) Key Laboratory of Primate Neurobiology; 200031 Shanghai, China.

[5] Ernst Strüngmann Institute (ESI) for Neuroscience in Cooperation with Max Planck Society; 60528 Frankfurt, Germany.

[†] Present address: International Institute for Integrative Sleep Medicine (WPI-IIIS), University of Tsukuba; Tsukuba, Ibaraki 305-8575, Japan.

[‡] Present address: Laboratory of Molecular Diagnostics, Department of Biotechnology, Inter-collegiate Faculty of Biotechnology, University of Gdańsk and Medical University of Gdańsk; 80-307 Gdańsk, Poland.

[§] Present address: Laboratory of Experimental Embryology, Institute of Genetics and Animal Biotechnology, Polish Academy of Sciences; 05-552 Warsaw, Poland.

[+] Senior authors.

[*] Corresponding author: julien.vezoli@inserm.fr.

Parkinsonian Monkey Rating Scale – PMRS

Clinical scoring was done independently by three trained experimenters at separate times throughout the day, 3-5 days per week using the Parkinsonian Monkey Rating Scale.[34-36] The PMRS contains a motor component, employing a previously published scale,[100] used to define the onset of the motor symptomatic period. The total score for freezing (0–3), resting tremor (0–3, for right and left side), rigidity (0–3), posture (0–2), bradykinesia (0–3), and ability to manipulate food (0–3, for right and left side) was used to frame injections (maximum total score of 23): a score below 5 defined the non-symptomatic state in premotor and recovery periods, and a score above 5 defined symptomatic periods. During the symptomatic post-MPTP period, all animals displayed the following clinical motor symptoms: freezing of gait, resting tremor, stooped posture, bradykinesia and difficulty to manipulate food at varying degrees. Only two cases displayed rigidity (cases 1 and 6), that was confirmed by clinical examination *i.e.*, arm manipulation. No further measure than observation was done to evaluate resting tremor and it might have been indistinguishable from postural tremor at rest;[61] however, resting tremor is a consistent feature in NHP after chronic low-dose MTP intoxication,[34, 38, 101, 102] while acute MPTP intoxication induces tremor only in some NHP species.[103, 104] Case 4 had a high-score post-MPTP (motor symptoms>15), characterized by tremor at rest and freezing. This motor-symptomatic state persisted during the 4 weeks prior to transplantation (Figure S3A). Case 6 had a high-score post-MPTP (motor symptoms>15), characterized by severe rigidity. Musculoskeletal pain is a recognized source of pain syndromes and discomfort in Parkinson's disease patients. We thus delivered regularly ketoprofen (Profenid), from week5 post-MPTP, in order to relieve suspected apparent musculoskeletal pain. Subsequently the clinical score for this animal progressively improved, due to amelioration on the rigidity scale and ability to manipulate food (from week5 to week10 post-MPTP, Figure S3A), but stabilized at week10 and remained largely symptomatic (score above 12) despite continuous treatment and disappearance of apparent musculoskeletal pain syndrome. Case 6 was transplanted at week23 post-MPTP.

Surgical procedure

We performed all transplantations bilaterally using 5µL Hamilton syringe and small trepanations over target sites. Stereotaxic coordinates were calculated from individuals T1 and T2 structural MRI images. We undertook sham-grafts (trepanation and injection of 5µL PBS without NPs) in cases 1 and 6 in order to control for behavioral and clinical effects due to the surgical act in itself. We placed sham-grafts (PBS-only) in posterior caudate and anterior putamen, using the same protocol as for NPs transplantation. The maximum number of injected cells per site was 1×10^6 and, the minimum number of injected cells varied between 3×10^5 for SN sites and 1.5×10^5 for striatum sites (Table S4). For all transplantations, the syringe was lowered 500µm further than the target position and then retracted to target; cells were then injected at a rate of 1µL/min after a 5min delay. Following a 3min delay after injection completion, the syringe was retracted slowly by 2mm and, another 2min delay was observed before full retraction. We assessed cell viability by trypan blue exclusion before and after injection by counting the remaining cells. Viability at both time points exceeded 95%.

Transplanted cells

We derived neural precursors (NPs) from a rhesus embryonic stem cell (RhESC) line stably expressing tau-GFP (LYON-ESC line).[39] NPs were obtained either as described previously,[97] and amplified in the presence of EGF and FGF2 (NPs, Figure S1A, upper panels) or after MS5 induced-neural differentiation,[98] followed by early midbrain DA differentiation (mDA-NPs, Figure S1A, lower panels). RT-PCR analysis returned similar marker expression profiles (except for the specific DA markers LMX1A and LMX1B which are not expressed in NPs; Figure S1B,D). Detailed protocols are further described below. Therefore, no a priori distinction between NPs derived from the two protocols was made for transplantation in different brain locations. To isolate NPs, RhESCs were manually selected and co-cultured on mitomycine C (MMC)-treated mouse embryonic fibroblasts (2.5×10^4cells/cm^2) in KO-DMEM medium containing Knockout serum replacement (KO-SR, 20%), non-essential amino acids (NEAA, 1X), and β-Mercaptoethanol (0.1mM), for 7-13 days.[97] Neuroepithelial-like cells that spontaneously emerged were selected manually (Passage 1 – P1) and cultured on gelatin-coated dishes, in DMEM/F12 medium containing N2 supplements (1X), non-essential amino acids (NEAA, 1X), β -Mercaptoethanol (0.1mM), Fibroblast Growth Factor2 (FGF2, 20ng/mL, Merck Millipore) and Epidermal Growth Factor (EGF, 20ng/mL, Sigma Aldrich). Amplification was done through mild trypsinization (Trypsin 0.025% - EDTA) followed by soybean trypsin inhibitor treatment. Medium was changed every other day. To induce early DA midbrain differentiation, RhESCs were seeded on MMC treated MS5 cells (day0). NPs emerging after 11-13 days were manually selected, dissociated with trypsin-EDTA, and cultured on matrigel-coated dishes (BD Biosciences, San Diego, http://www.bdbiosciences.com) in Neurobasal medium containing B27 supplement (without Vitamine A, 1X), 5µg/mL bovine fibronectin, FGF2 (20ng/mL). From day6-8, we added the dorso-ventralizing factors: FGF8 (100ng/mL), Sonic hedgehog (SHH, 200ng/ml) and FGF20 (2ng/mL) as described previously.[98] To induce differentiation into mature DA neurons, Wnt3a (100ng/mL) was added to the culture medium from day11 to day24. From day24 to day45, mDA-NPs were cultured in the presence of Brain Derived Neurotrophic Factor (BDNF, 20ng/mL, Sigma Aldrich), Glial cell line-Derived Neurotrophic Factor (GDNF, 20ng/mL, R&D Systems), Transforming Growth Factor β2 (TGFβ2, 2ng/ml, R&D Systems), dibutyryl cAMP (0.5mM, Sigma Aldrich), Neurotrophin-3 (NT3, 20ng/mL, Sigma Aldrich), Wnt5a (100ng/mL) and Ascorbic Acid (AA, 0.2mM, Sigma Aldrich). To induce neuronal differentiation, NPs were trypsinized (Trypsin 0.025% - EDTA, 0.1g/L), replated (2.5×10^4cells/cm^2) into Matrigel-coated dishes, and cultured in DMEM/F12 medium supplemented with N2 (1X) and FGF2 (10ng/mL). After 7 days, the medium was changed to DMEM/F12 medium mixed with Neurobasal medium (1:1) supplemented with N2 (0.5X), B27 (0.5X) and FGF2 (5ng/mL). After an additional 7 days in these conditions, medium was switched to Neurobasal medium supplemented with B27 (1X). To induce glial differentiation, NPs were cultured in DMEM/F12 medium supplemented with 10% Fetal Bovine Serum (FBS, HyClone, Perbio) for 7 days. All reagents were from Invitrogen, unless otherwise indicated. For cell transplantation, we dissociated NPs into single cells using trypsin-EDTA to prepare a suspension at a concentration of 0.3-2.0×10^5 cells/µL and, injected 5µL per site. Grafted NPs express

specific neural markers (Figure S1A-B,D) and showed *in-vitro* potential to fully differentiate along glial and neuronal pathways (Figure S1C), including mature DA neurons (Figure S1C). Despite the fact that RhESC-derived NPs were transplanted in Macaca fascicularis, we choose to not carry out immunosuppressive treatment as previously done in other Xenografts studies,[105,106] and in preclinical studies assessing the potential of human cell grafts. Relying on the extensive sharing of MHC alleles,[107] and hybridization evidence,[108] we anticipated low immune response and decent survival.[109] We avoided the inconvenient of immunosuppressive therapy in Parkinsonian macaques (e.g., increased risk of infectious complications) by assuming limited immunogenicity of transplanted cells differentiated from RhESC into the cynomolgus macaque brain.

Cognitive behavior – detour task

Cognitive performance was monitored 3-5 days a week using a previously described behavioral test.[34, 36] Briefly, performance on the 'detour task' was evaluated by the percent of successes (retrieval of reward on the first reach, over the total number of trials) and errors (barrier hits, over the total number of responses observed - there could be several responses per trial except in the case of success). Errors due to motor impairments were classified as such and further discarded from the performance evaluation, so as to avoid confounds due to the symptomatic state of the animal. The performance on the 'detour task' depends on the integrity of frontal cortex, the dopaminergic system, the dopaminergic innervation of frontal cortex,[110] and has been used to assess the impact of lesion of the nigrostriatal axis on cognitive performance as well as potential therapy for cognitive impairments.[111-113] For inter-individual comparison, percent errors and success were, for each case, first converted in percent change by the average control value and range normalized between 0 and 1 by maximum and minimum score (normalized contrast, Figures 3A, S3B).

Circadian rhythm follow-up

Animals were maintained under an LD cycle (12h light: 12h dark) of approximately 450–500 lux during the light phase and 0 lux during the dark phase. Locomotor activity was continuously recorded in 1 min intervals throughout the entire duration of the study using passive-infrared motion detectors mounted above each animal's cage. The motion captors were connected to a computerized data acquisition system (Circadian Activity Monitoring, INSERM, France).[114] Data were analyzed using the Clocklab software package (Actimetrics, Evanston, IL, USA). We previously used Chi-squared periodogram method (Table S1) to calculate the period of behavioral rhythm which is defined as the time elapsed for one complete oscillation or cycle (the distance in time between two consecutive peaks or troughs of a recurring rhythm). The stability and the consistency of the rhythm are reflected by the value of the periodogram amplitude which corresponds to the zenith of a periodogram curve. Non-parametric circadian rhythm analyses (NPCRA) were used to estimate the strength and fragmentation of rest-activity rhythms.[34, 35] These included measurements of inter-daily stability (calculated as the ratio between the variance of the average 24-hour pattern around the mean and the overall variance and gives an indication of the consistency of day to day activity or the strength of coupling to the LD cycle), intra-daily variability (calculated as the ratio of the mean squares of the difference between successive hours and the mean squares around the grand mean and gives an indication of the frequency of transitions between rest and activity periods, corresponding to the fragmentation of the rhythm) and, relative amplitude (ratio between acrophase and nadir of the rhythm, representing the ratio between activity amplitude in light and dark phases). When the rest-activity rhythm is stable, inter-daily stability is high, the intra-daily variability is low and relative amplitude is high.[34, 35] MPTP intoxication induced a reduction of relative amplitude and an increase of rhythm variability and fragmentation; however, NPCRA revealed high variability across cases preventing post-graft group comparison on those markers (data not shown). For each case, Ligh/Dark ratio was first converted in percent change by the average control value and range normalized between 0 and 1 by maximum and minimum values (normalized contrast, Figures 3A, S3C).

Dopamine function imaging – [11C]-PE2I and [18F]-FDOPA

To evaluate the in-vivo DA function, we acquired images from Positron Emission Tomography (PET) scans using (E)-N-(3-iodoprop-2-enyl)-2beta-carbomethoxy-3beta-(4'-methylphenyl)-nortropane labeled with carbon 11 ([^{11}C]-PE2I) at different intervals throughout the protocol as

described previously,[36] in cases 1, 2, 3 and 6 and in cases 1, 3 and 6 after grafts at different delays (see Figures S4A). [^{11}C]-PE2I specifically binds with high affinity and selectivity to DA transporters (DAT, Ki = 17nM) and is considered to provide an index of the integrity of the DA pathway that has been used for Parkinson's disease diagnosis.[115-117] Additionally, we used images from PET scans using L-3,4-dihydroxy-6-(^{18}F)fluorophenylalanine ([^{18}F]-FDOPA, an analog of the DA precursor L-DOPA) to evaluate the central dopaminergic function of pre-synaptic neurons, i) just before grafts (during stable expression of typical motor parkinsonian symptoms *i.e.*, Stage II, the symptomatic period) and, ii) after grafts at different delays (Figure S4B). We performed [^{11}C]-PE2I and [^{18}F]-FDOPA PET scans with an ECAT Exact HR+ tomograph (Siemens CTI), in 3D acquisition mode, covering an axial distance of 15.2cm. The transaxial resolution of the reconstructed images was about 4.1mm full-width and half maximum in the center. We acquired transmission scans with three rotating 68Ge sources to correct emission scans for the attenuation of 511 keV photonrays through tissue and head support. Full procedure for PET-scans acquisition, modeling and ROIs definition for regional assessment of [^{11}C]-PE2I changes have been published.[36] Fluoro-DOPA was labeled with ^{18}F-fluoride (cyclotron–produced isotope, half-life = 109 min). Specific radioactivity was 3.27 ± 1.3 mCi/µmol. Radiochemical and chemical purity of produced [^{18}F]-FDOPA (as determined by HPLC) was above 99%. After anesthesia was induced and head secured with a MRI-compatible stereotaxic frame (Kopf, CA, USA) to reduce variability in the measure, a cannula was inserted in the femoral vein. [^{18}F]-FDOPA was injected as a bolus over a 4 s period followed by a saline flush. Radioactivity was measured in a series of 26 sequential time frames of increasing duration (from 30sec to 5min; total time 90min). PET modeling and regional assignment of PET changes were done as previously described,[36] and applied to F-DOPA measurement.[63] Statistical positive (shown in Figure 3B) and negative (shown in Figure S10C, right) differences were determined from 2.5th and 97.5th percentile boundaries of voxel-level distribution statistics of all brain ROIs as described previously,[36] and illustrated in Figure S10C, left.

Immuno-histological procedures

Deep anesthesia was induced with Ketamine after premedication with chlorpromazine hydrochloride (Largactil) followed by a lethal dose of pentobarbital sodium (Vibrac, 100 mg.kg-1, i.p.; confirmed by complete loss of corneal reflex) before animals were transcardially perfused with saline (0.9% with procaine) and 4% paraformaldehyde in phosphate buffer (PB). Cryoprotection was ensured by sucrose gradients (10-30%) perfusion post-fixation. Brains were removed, kept in cryoprotecting liquid overnight and coronal 50µm thick sections cut on a freezing microtome and collected serially. Sections were processed together with equivalent sections from control animals for Tyrosine Hydroxylase (TH; Millipore, #MAB318) and DAT (Millipore, #MAB369) immunochemistry visualized with 3,3-diaminobenzidine, in Ni^{2}+H202 (0.5-1.0%). All sections were washed, mounted, dried, and dehydrated in increasing gradients of ethanol, cleared in toluene, mounted with mounting medium and cover slipped. Immunostainings of cultured cells were done according to previously published protocol.[39] Briefly, cells were fixed with 2% PFA in PB at 4°C for 1 hour, and permeabilized in Tris buffered saline (TBS) with 0.1% Triton X-100 (3×10min). Non-specific binding was blocked with 10% normal goat serum (NGS) for 20min at room temperature (RT). Cells were incubated overnight at 4°C with primary antibodies diluted in Dako diluent. After three rinses in TBS, cells were exposed to secondary antibodies for 1 hour at RT followed by nuclear staining with DAPI (1/10,000) for 3min. After three rinses in TBS, coverslips were mounted on slides, and observed under confocal microscopy. For *post-mortem* immunofluorescent staining of brain slices, sections were washed six to ten times in Tris buffered saline (TBS) and permeabilized in Triton X-100 (0.5%, SIGMA, #T9284). Nonspecific binding was blocked with 10% NGS or donkey serum (Goat: Invitrogen; #16210064; Donkey: SIGMA #D9663) for 45 min at room temperature. Sections were incubated for three days at 4°C, with primary antibodies diluted in Dako antibody diluent (Dako #S3022) supplemented with 0.5% Triton X-100. They were then exposed to secondary antibodies and DAPI (1/10,000; Invitrogen #D1306) for 2 hours at RT. Sections were mounted on slides and examined using a confocal microscope (Leica TCS SP5). Sections were regularly processed for GFP immunostaining

to detect grafted sites extent (every five sections *i.e.*, every 250µm). The list of antibodies used presented in Table S6. For semi-quantitative RT-PCR analyses, total RNA was prepared with a QIAGEN RNeasy kit (QIAGEN, Valencia, CA, http://www.qiagen.com). Standard reverse-transcription reactions were performed with 1µg of total RNA primed with random primers using SuperScript II first strand synthesis system, according to the manufacturer's recommendations (Invitrogen). Oligonucleotide sequences are given in Table S7.

Quantification procedures

Stereological analyses were used to estimate the total number of TH-positive neurons in the ventral midbrain. A light microscope (Leica DM6000) equipped with a digital camera, a motorized x-y stage and a Z-axis indicator was controlled by the stereological software (Mercator, Explora Nova). Sections stained for TH were counterstained with Cresyl Violet (Figure 2B). We first draw contours of SN and VTA at low magnification based on a stereological atlas.[118] The unbiased stereological estimation of the total number of DA neurons in the SN and VTA was then achieved using the optical fractionator principle (Mercator, Explora Nova). Combining the optical dissector with a fractionator sampling scheme, this technique is not affected by tissue volume change. TH-positive cells were counted using a 20× oil-immersion objective (0.70 N.A.) in one out of every sixteen sections *i.e.*, every 800µm through the rostrocaudal extent of ventral midbrain. The stereology software placed counting frames on a regularly translated sampling grid formed of 350 x 350µm squares within the chosen region of interest and controlled the motorized x-y stage of the microscope in order to control the scan of the entire brain region. Nuclei of immunolabeled cells that fell inside the counting frame and did not contact the exclusion lines were counted (CE for controls between 0.04 and 0.056). We measured the length of processes emanating from the graft core by measuring the length of tau-GFP positive projections (ImageJ, NeuronJ plugins, http://imagej.nih.gov/ij/), presented in Figure 5D. We estimated each graft volume through a 3D reconstruction process using contours (ExploraNova, Mercator, http://www.exploranova.com/products/mercator/), presented in Figures 5, S5. We determined the average cell density by i) counting nuclei stained with ToPro3 or DAPI of tau-GFP$^+$ cells in four random locations per confocal plane *i.e.*, confocal optical section, in several slices at different levels of the graft core to return cell density per mm^3 and ii) by scaling those counts to the estimated graft volume in mm^3 to return estimated cell number per graft. Finally, we calculated survival rate (Table S3) according to the number of transplanted cells per site (Table S4). To estimate the proportion of TH$^+$, MAP2$^+$ and GFAP$^+$ areas in each grafted site, confocal images were acquired at random locations of the tau-GFP$^+$ grafts. We measured TH$^+$, MAP2$^+$, GFAP$^+$ and tau-GFP$^+$ areas on binary images of the corresponding channel (ImageJ, http://imagej.nih.gov/ij/) and determined the co-expression ratios of TH$^+$/tau-GFP$^+$ MAP2$^+$/tau-GFP$^+$ and GFAP$^+$/tau-GFP$^+$ on at least 4 z-sections per grafted site. For semi-quantitative immunohistochemistry, three age and weight matched Macaca fascicularis served as controls. We evaluated the extent and amplitude of the nigrostriatal lesion, excluding graft locations as previously described.[36, 119] The mean optical density (O.D.) of TH and DAT immunopositive regions were computed on at least 5 sections per animal (5-20) from 8bit images, normalized and compared with controls (immunostaining with 3,3'-diaminobenzidine – DAB revealed in the same bath).[36] In Figure 2A, composite images of control and MPTP animals has been enhanced for brightness and contrast, for illustration purpose. Importantly, decreased levels of striatal DAT O.D. compared to controls were consistent with the decrease of striatal DAT binding as evaluated *in-vivo* by PET.

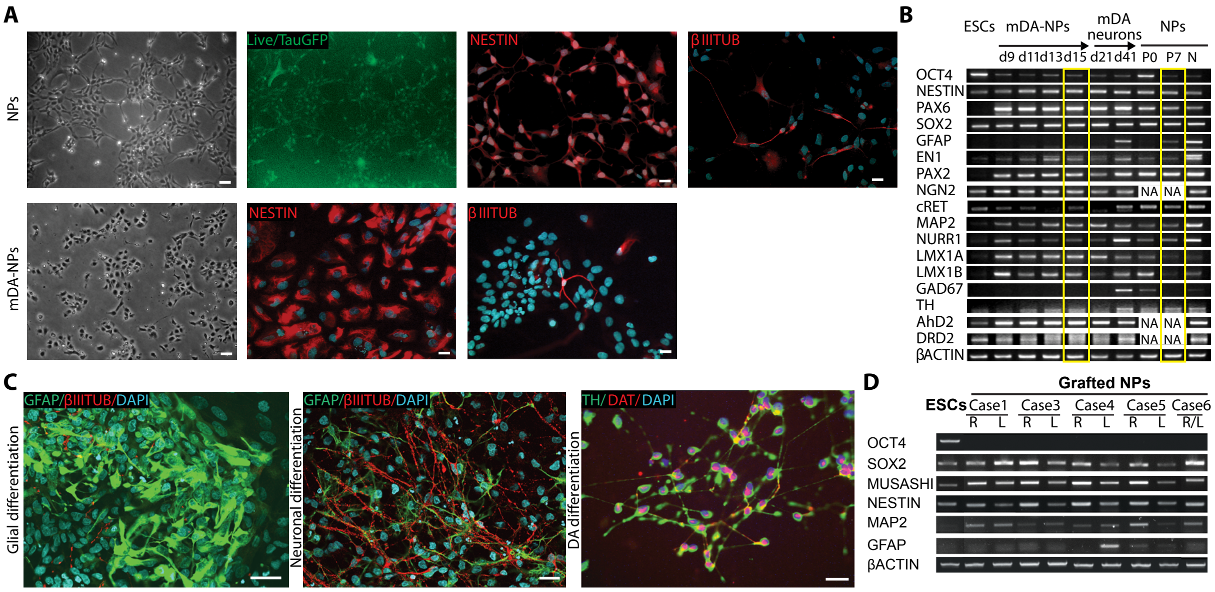

Figure S1: Characterization of grafted cells

(**A**) NPs derived from the rhesus Embryonic Stem Cells (RhESCs, LYON-ES1 line) stably expressing tau-GFP. Upper panels from left to right, NPs at Passage 4 (P4), phase contrast and corresponding live fluorescence images (scale bar 50µm) and immunostainings for NESTIN and βIII-TUBULIN (scale bars 20µm). Lower panels from left to right, mDA-NPs isolated from rosettes (P1d17), phase contrast image and immunostainings for NESTIN and βIII-TUBULIN (scale bars 20µm). (**B**) From left to right, RT-PCR analysis of specific gene markers in ESCs, during differentiation into mDA-NPs and mDA-neurons, and in NPs (P0-P7 and N for NP-derived neurons). For example, markers specific for ESCs (OCT4, SOX2), NPs (NESTIN, SOX2, PAX6), glial (GFAP), neuronal (MAP2), DA progenitors (LMX1A, LMX1B, NURR1), or DA neurons (TH, AhD2, DRD2). Yellow rectangles indicate stages at which NPs were transplanted. (**C**) Immunofluorescence staining for βIII-TUBULIN and GFAP upon glial (left) and neuronal (middle) differentiation of NPs (scale bars 50µm) and, staining for DAT and TH of mDA-neurons (d40) (scale bar 10µm). (D) RT-PCR analysis showing comparison of marker genes in ESCs and NPs, for each case, at the time they were transplanted in right (R) and left (L) hemisphere. d, day; βIIITUB; βIII-TUBULIN.

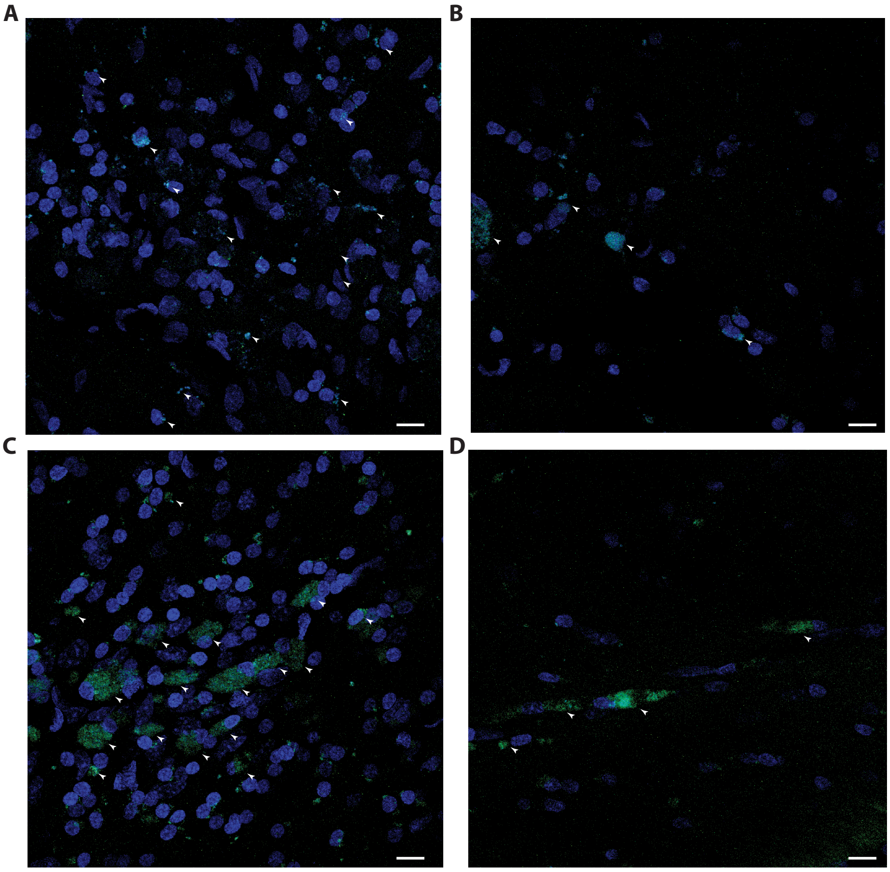

Figure S2: Grafted NPs did not survive in the No Graft Survival group
(**A-D**) tau-GFP (green) and ToPro3 (blue) immunolabeling of SN tissue at grafted locations in case 6 showing only cell debris (arrowheads) surrounded by weak and diffuse tau-GFP staining. Scale bars, 10µm.

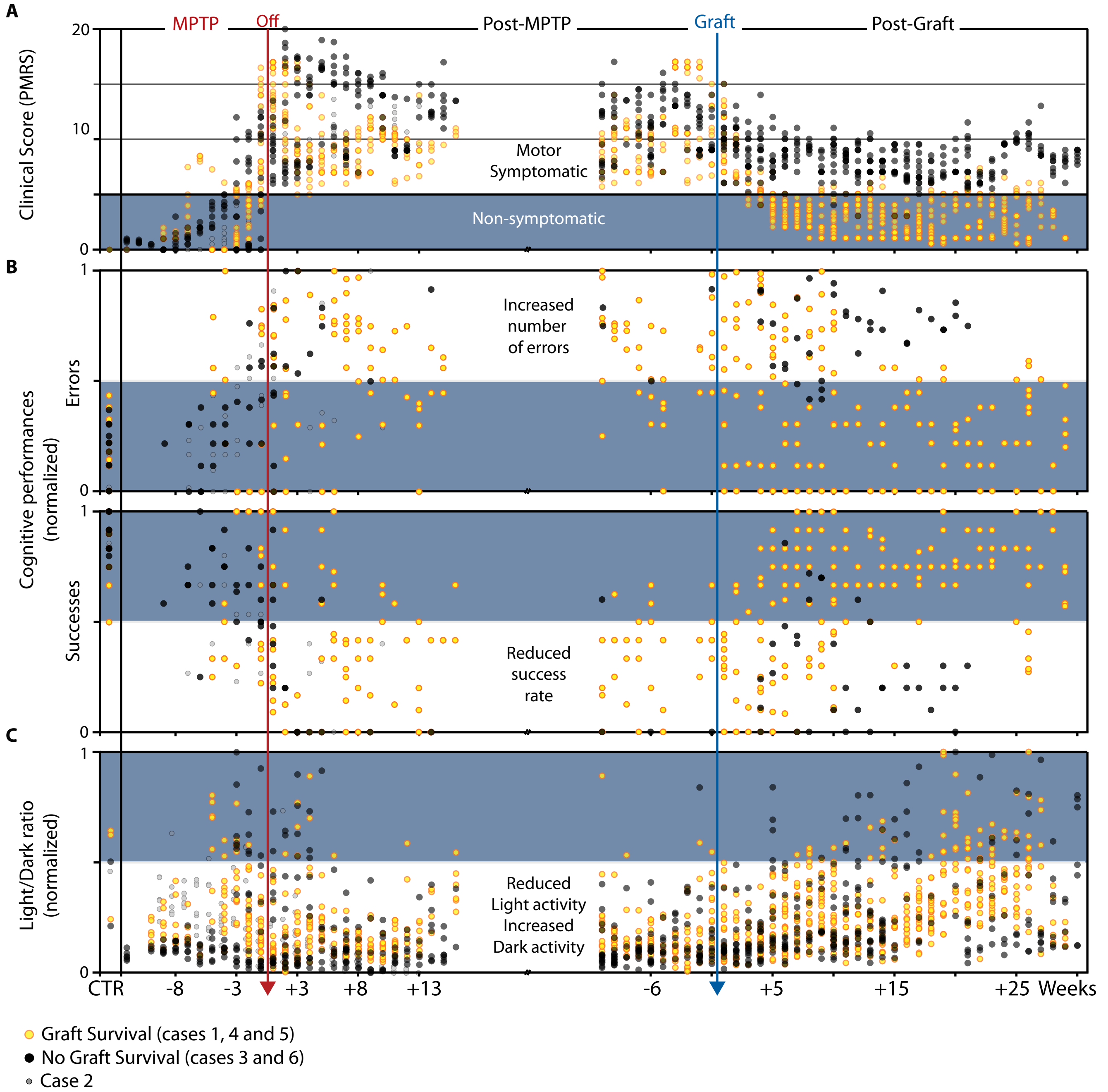

Figure S3: Individual follow-up of behavioral and clinical markers

Weekly values of motor and non-motor cognitive & circadian behavioral markers for all cases as groups (legend down left) after alignment to MPTP offset (red line) and NP grafts (blue line). (**A**) Clinical motor score, a score ≥ 5 (clinical threshold) indicates symptomatic state corresponding to clinical diagnosis for PD patients. (**B**) Cognitive performances (Errors – up and Successes – down at the object retrieval detour task, see Methods for details), normalized by min-max per case. (**C**) Ratio of spontaneous locomotor activity during the light and dark periods (normalized contrast with Light/Dark ratio in control conditions per case, see Methods). Note in the Post-MPTP / Pre-graft period, the reduced number of data points for the No Graft Survival group are due to an elevated motor score (A) and its impact on the ability to perform the task i.e., a majority of trials contained motor errors during performance of the task and were therefore discarded from the evaluation (see Supportive Information Methods).

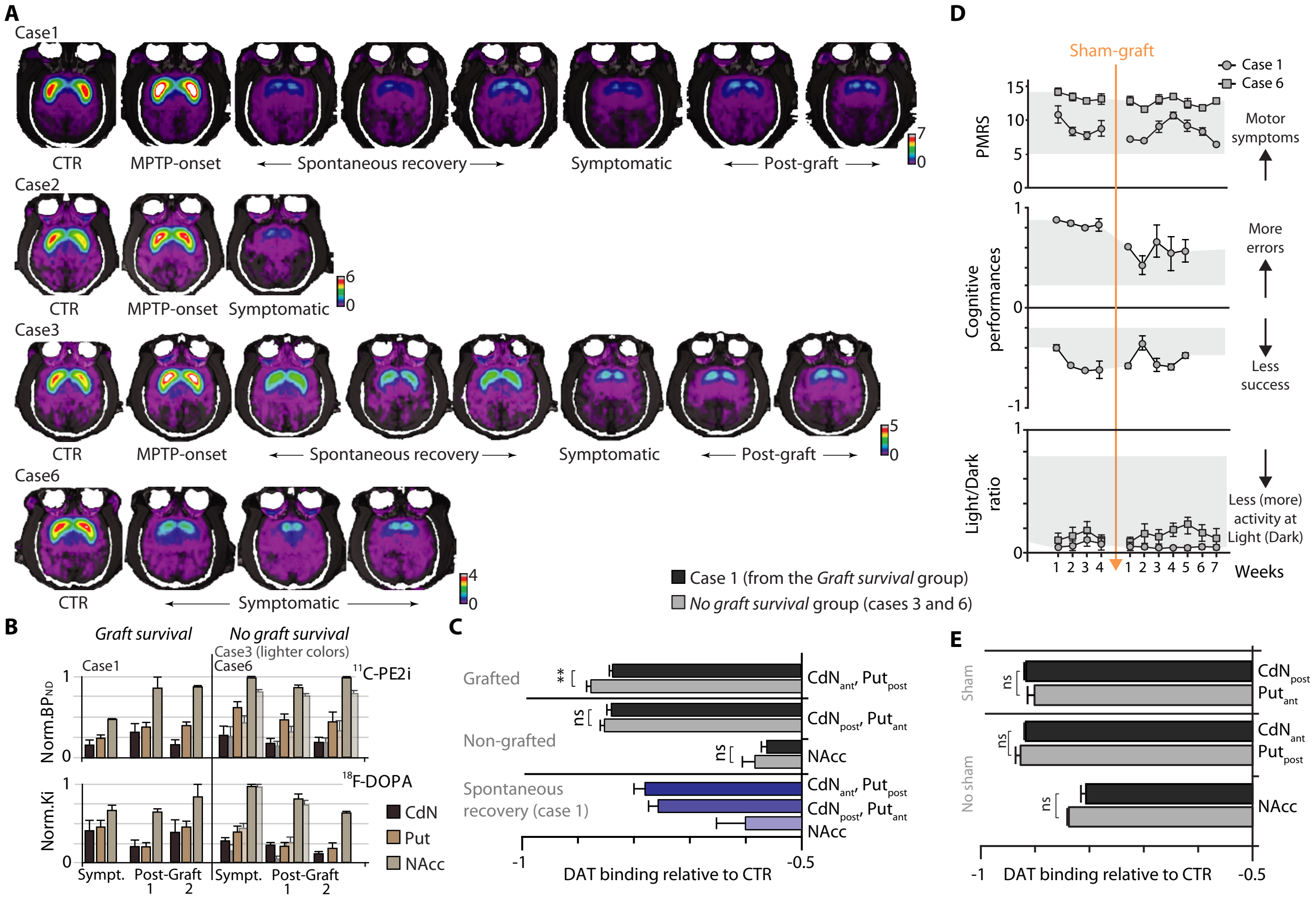

Figure S4: Functional follow-up of striatal DAT binding and Sham-grafts consequences on behavioral and functional markers

(**A**) PET-scan images reconstruction after alignment to individual MRI showing progression of DAT binding from CTR until post-Graft periods. Color scale for BPND. (**B**) Functional markers of nigrostriatal innervation (normalized BPND of 11C-PE2i) and DA function (normalized Ki, uptake of 18F-DOPA) in case 1 and cases 3 & 6) during expression of stable symptoms (Symptomatic – Sympt.) and following transplantation (Post-Graft, at 2 time points post-graft separated by a minimum of 10weeks, 1: Symptomatic i.e., PMRS ≥ 5 in all cases and 2: Recovery in case 1 and Symptomatic in cases 3 and 6). Min-max normalization per case. Means ± SE. Note that 18F-DOPA was measured only once post-graft for case 3. (**C**) Percent change in Striatal DAT binding in Grafted and Non-grafted regions for case 1 (from the Graft Survival group in black) and the No Graft Survival group (cases 3 and 6, in grey). Purple, Striatal DAT binding following induced spontaneous recovery (case 1). Two-sided Wilcoxon rank sum test, Bonferroni correction for multiple comparisons ** $P < 0.01$, $Z = 2.92$. (**D**) Sham-grafts, performed in cases 1 and 6, show no effect on motor nor on non-motor circadian symptoms. Case 6 remained too affected to perform the task, and cognitive performances of case 1 remained strongly and stably impaired compared to CTR before and after sham surgery. From top to bottom, clinical motor score – PMRS, cognitive performances (errors – up, successes – down, range normalized to control performances, -1 for success) and Light/Dark ratio. Means ± SE. (**E**) PET-scans of 11C-PE2I (relative change compared to CTR) show no difference in DAT binding following Sham-graft between case 1 vs. case 6 (Two-sided Wilcoxon rank sum test).

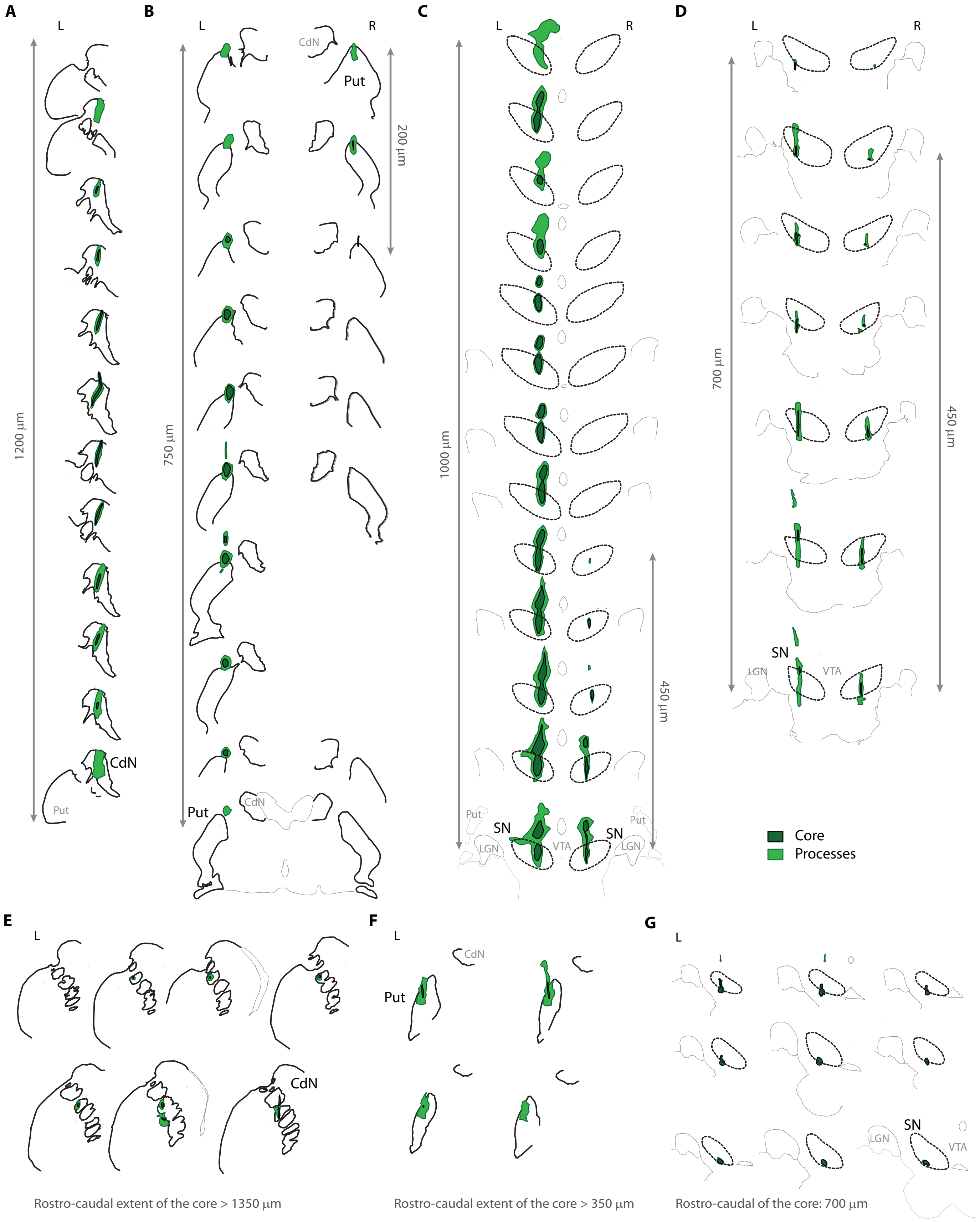

Figure S5. Post-mortem localization of grafted NPs
Digital reconstructions of post mortem grafts' core in case 5, (**A**) left anterior CdN, (**B**) bilateral posterior Put and (**C**) bilateral SN; in case 1 (**D**) bilateral SN, (**E**) left anterior CdN at the limit with anterior Put and (**F**) left posterior Putamen; in case 4 (**G**) left SN. Same conventions as in Figure 5. CdN: caudate nucleus; Put: putamen; SN: substantia nigra; VTA: ventral tegmental area; LGN: lateral geniculate nucleus.

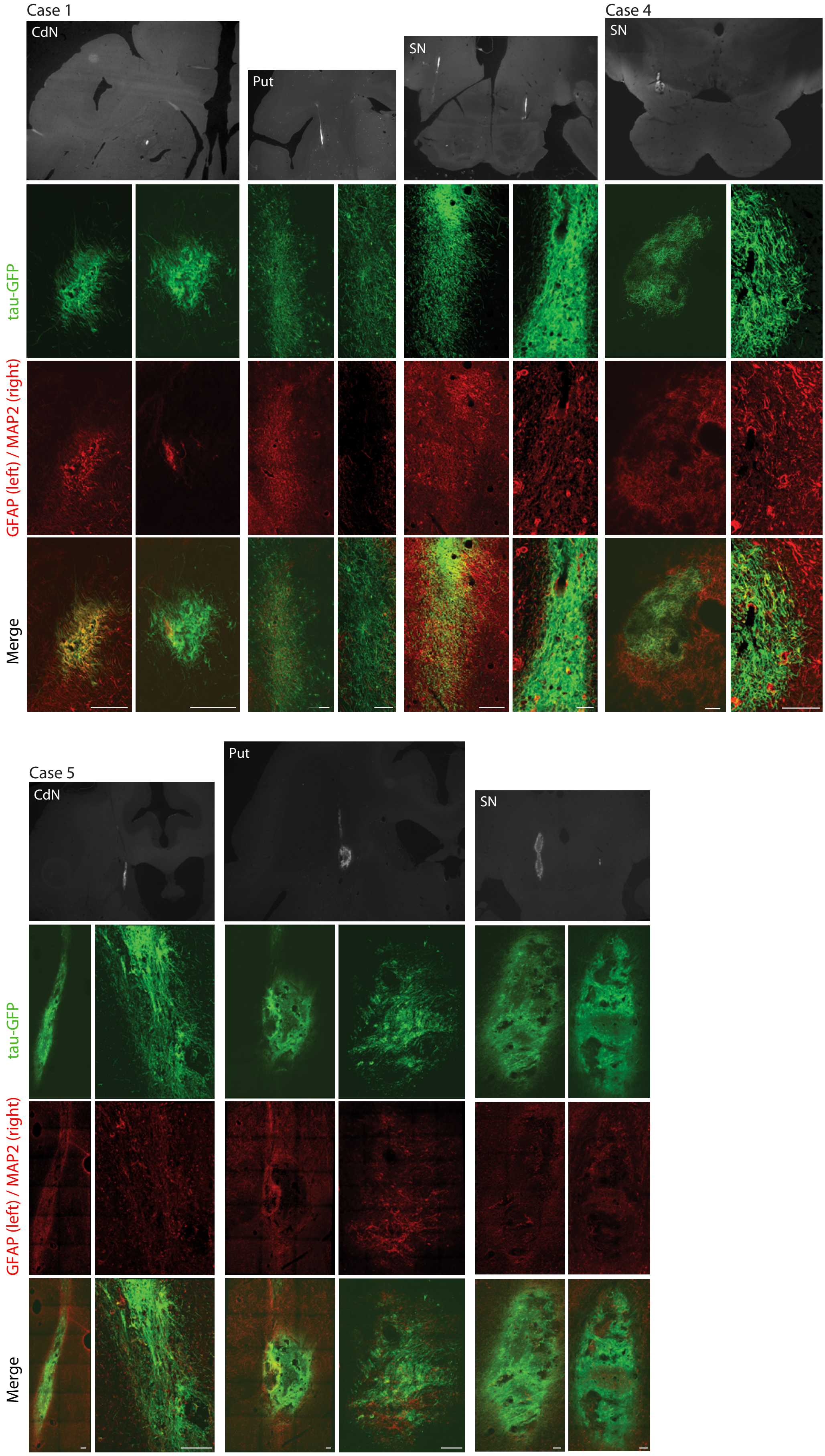

Figure S6: Post-mortem localization and characterization of transplanted NPs
Photomicrographs of tau-GFP+ grafts and immunofluorescence labeling at different sites for cases 1, 4 and 5. Scale bars, 100µm. Grayscale images from fluorescent microscope (filter 488nm, LeicaM165 FC).

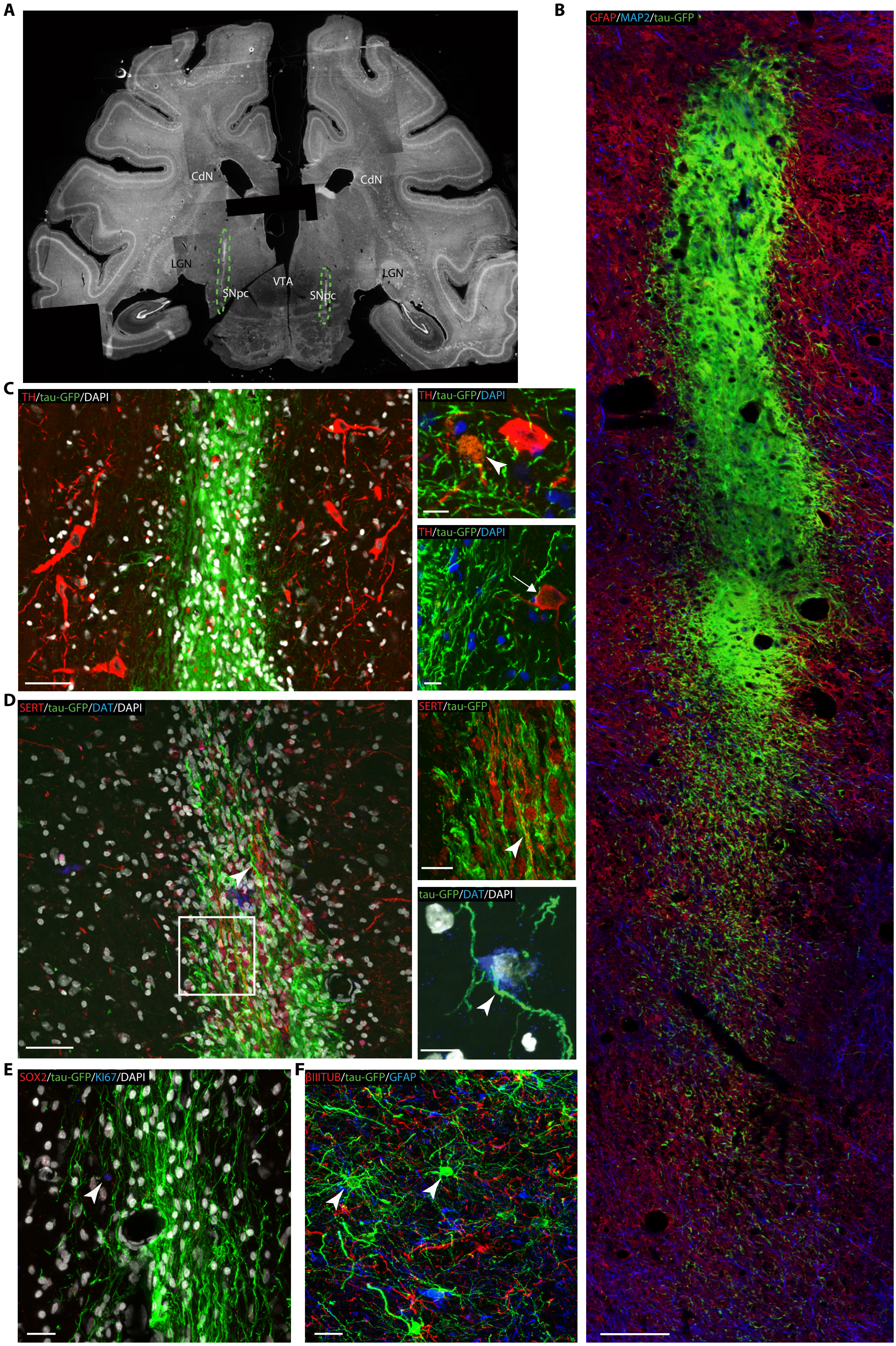

Figure S7: Grafted NPs in case 1

(**A**) Photomicrograph from fluorescent microscope acquisitions (tau-GFP+ in white, filter 488nm, LeicaM165 FC) showing location of NPs in nigral transplant (green). (**B**) Closer view, confocal acquisition at an equivalent section. (**C**) Grafted cells (tau-GFP+) in SN tissue surrounded by surviving host TH+ cells, some of which co-express TH+ (arrowhead, top-right panel) and seems to contact host aminergic cells (arrow, bottom-right panel). (**D**) Photomicrograph at SN site showing grafted cells processes co-expressing SERT+ (frame enlarged in top-right panel, arrowhead) and surrounding host DA cell (arrowhead, DAT+, bottom-right panel). (**E**) Host cell expressing KI67 (arrowhead). (**F**) Grafted cells showing glial morphology. Scale bars: B 100µm; C 10µm; D left 50µm, top-right 10µm, bottom-right 20µm; E-F 20µm.

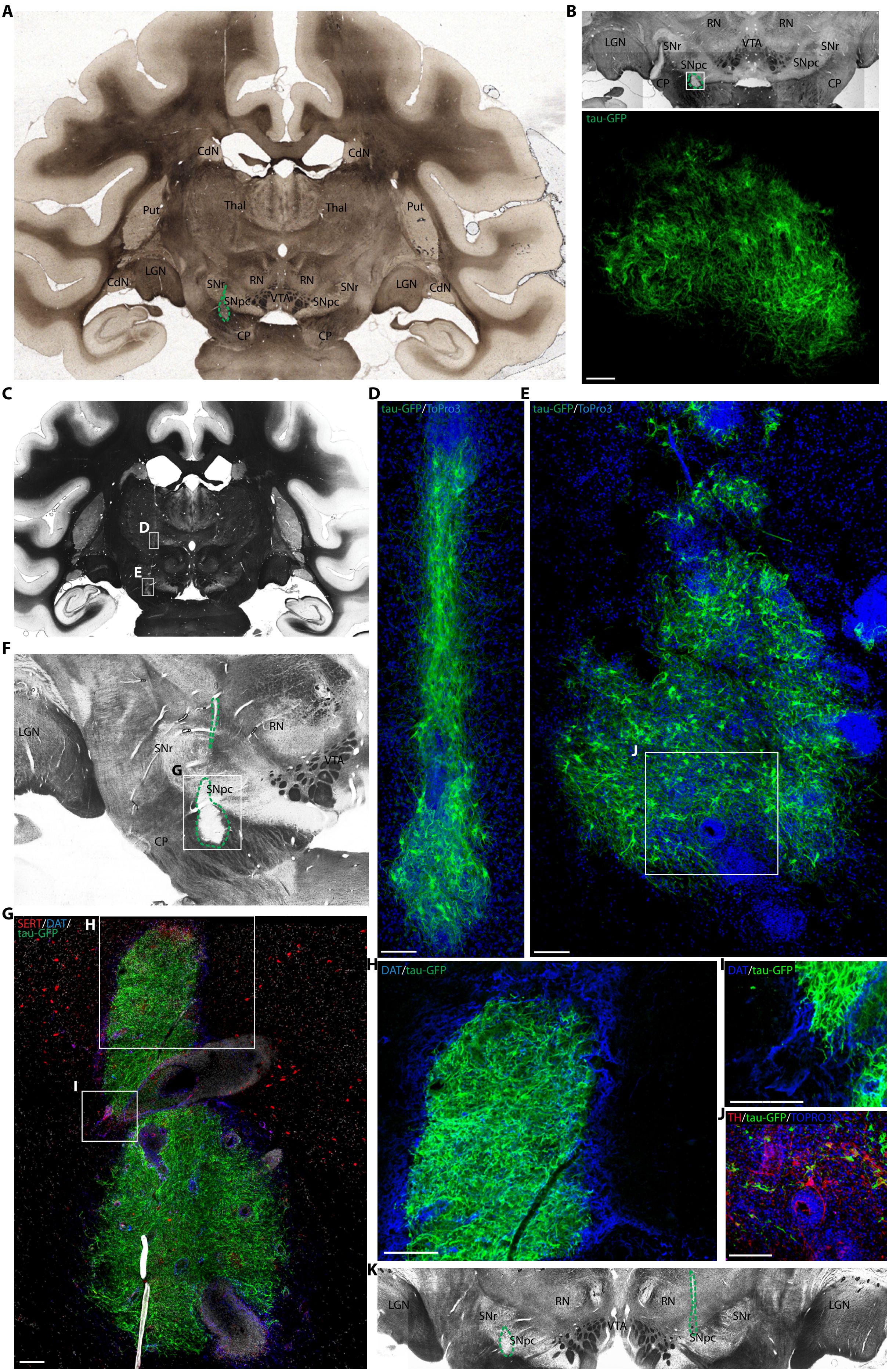

Figure S8: Grafted NPs in case 4

(**A**), (**B**), (**C**), (**F**), (**K**) Grayscale images from white-field microscope showing location of NPs in nigral transplant (green). (**B**) Bottom panel show tau-GFP+ cells framed in top panel. (**C**) Framed regions shown in (**D**) and (**E**). (**G**) Framed region in (**F**) showing concentration of DAT+ processes on the graft core and surroundings, shown at higher magnification in (**H**) and (**I**). (**J**) Host cells aggregate surrounded by a host TH+ cell, region framed in (**E**). Scale bars: B, E, G-J 100µm; D 50µm.

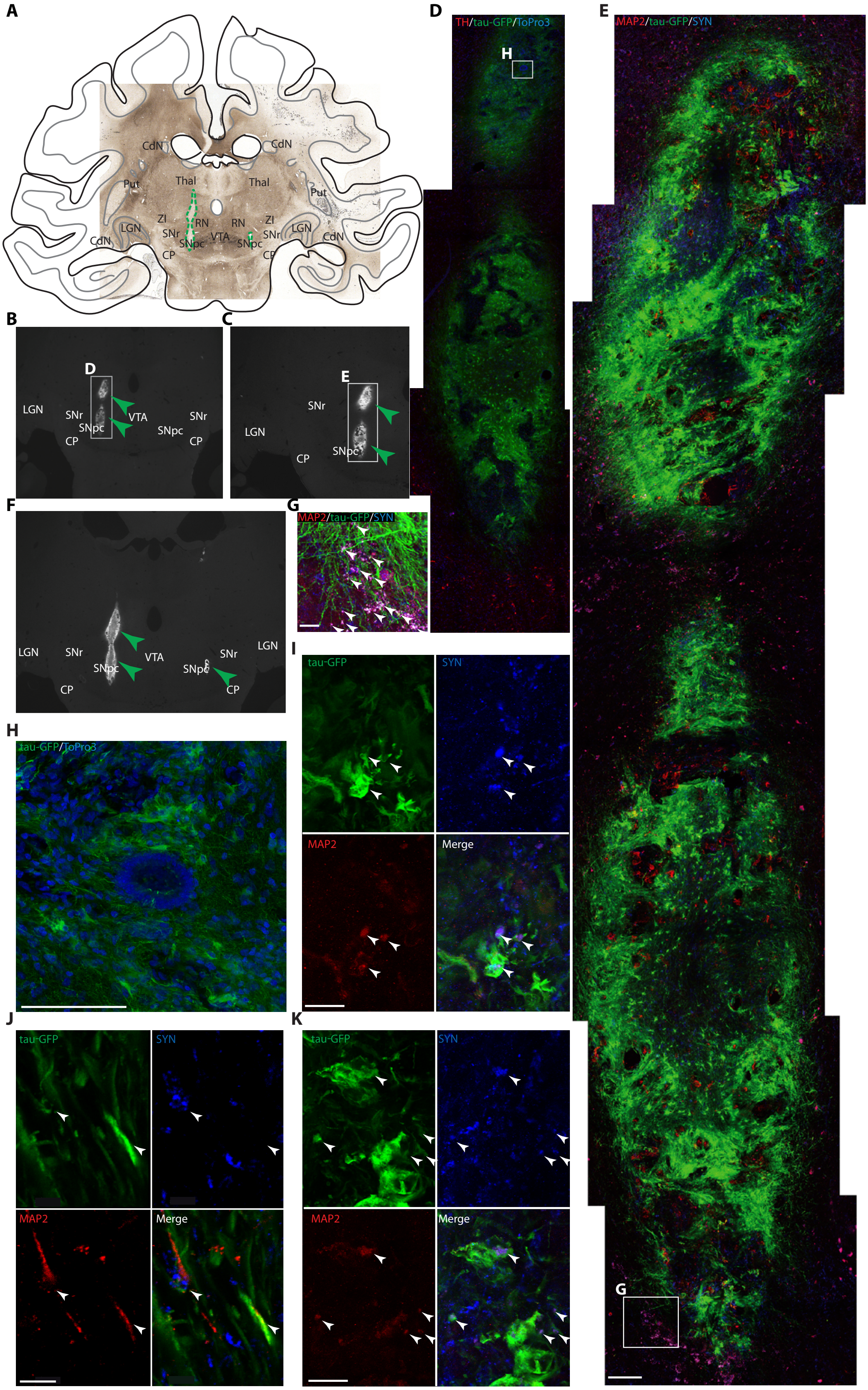

Figure S9: Grafted NPs in case 5

(**A**) Grayscale images from white-field microscope showing location of NPs in nigral transplant (green), drawings of the complete brain region from an adjacent section. (**B**), (**C**) and (**F**) Photomicrograph from fluorescent microscope acquisitions (tau-GFP+ in white, filter 488nm, LeicaM165 FC). (**D**) and (**E**) Framed regions in, respectively (**B**) and (**C**). (**G**) Framed region in (**E**) showing accumulation of SYN+ and MAP2+ where tau-GFP+ processes extend outside the core (white arrowheads. (**H**) Framed region in (**D**) showing host cell aggregate inside the graft-core. (**I**), (**J**), (**K**) Grafted cells (tau-GFP+) co-localized with SYN+ and MAP2+. Scale bars: E 100µm; G 25µm; H 100 µm; I-K 5 µm.

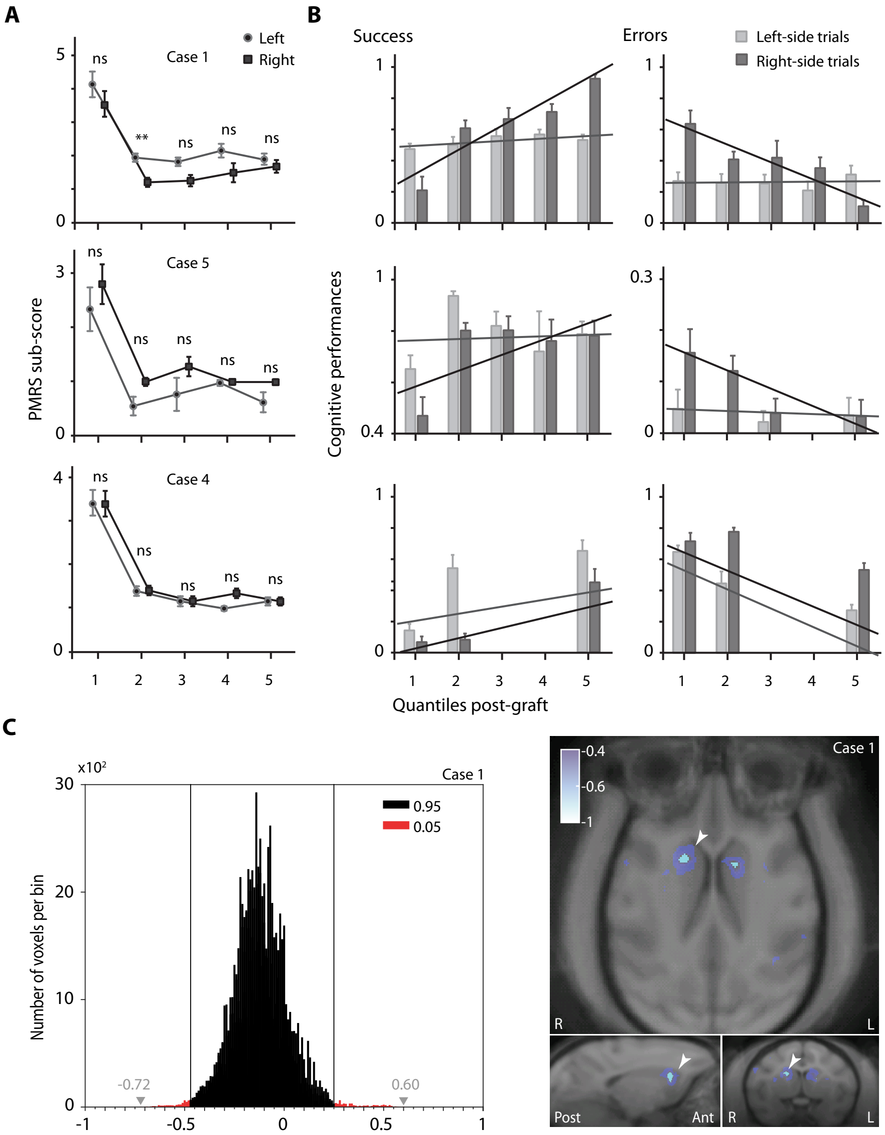

Figure S10: Hemispheric differences in graft-induced recovery

(**A**) Evolution of clinical sub-score in quantiles post-graft. Average sum of clinical score for resting tremor, ability to manipulate food, lower and upper limb movements for case 1 (top), case 5 (middle) and case 4 (bottom) that was scored separately for left (in grey) and right limbs (in black). Note that no clear difference could be related to NPs survival in left vs. right hemisphere. Case 1 presented unilateral survival in posterior Put and bilateral survival in SN whereas case 5 presented unilateral survival in SN and bilateral survival in posterior Put. Case 4 is presented for completeness as no NPs were grafted in the Put (see Table S3). ** P < 0.001, ns P > alpha (Bonferroni corrected alpha = 0.01, two-sample t-test assuming unequal variance). (**B**) Cognitive performances evolution in quantiles post-graft. Lines represent linear regression of average performances per quantile for left (grey lines) and right (black lines) trials separately. Recovery is characterized by an increase in Success and a decrease in Errors. Note the time-course asymmetry of recovery for cases 1 and 5 (top and middle row) and parallel time-course for case 4 (bottom row) when comparing left vs. right trials. Cases 1 and 5 presented unilateral survival whereas case 4 presented bilateral survival in the anterior CdN (see Table S3). Mean±SE across quantiles. (**C**) The distribution of difference values of voxel-level BPND Pre- vs. Post-graft, over all brain ROIs was used to calculate the 2.5th and 97.5th percentile boundaries (case 1, vertical lines, -0.48 and +0.26 respectively). Grey arrowheads indicate min and max difference bins. Corresponding statistical thresholding of negative difference (right panel), note the minor but significant decrease in right anterior CdN (arrowheads). Positive difference shown in Figure 3B.

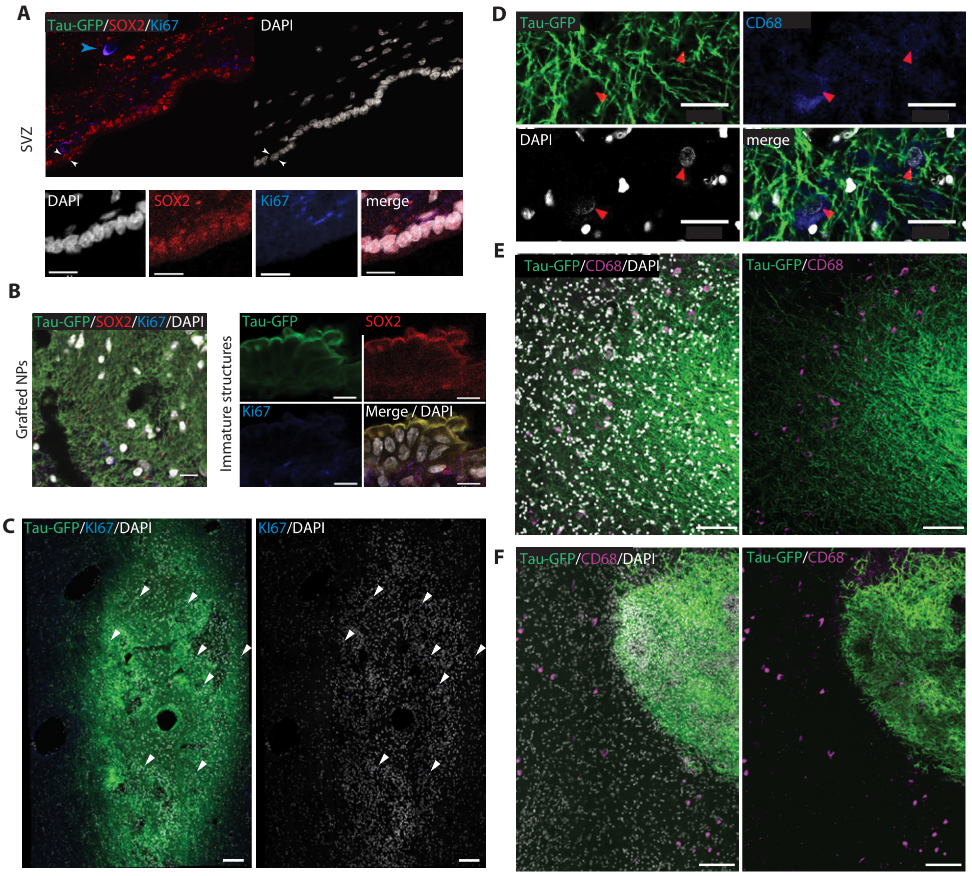

Figure S11: NPs show no overgrowth after transplantation and induce minor immune reaction
(**A**) Typical sub-ventricular zone section (SVZ, white arrowheads, case 5) displaying some proliferation (Ki67+, blue arrowhead) and NP (SOX2+) markers, scale bar 20µm. (**B**) Case 5. Left, post-mortem immunostaining showing that no more NPs remain after graft (SOX2-) from the tau-GFP population (in green) which neither shows sign of proliferation (Ki67-); scale bar 13µm. Right, section at the level of a structure exhibiting an immature morphology which was however negative for SOX2- and Ki67-; scale bar 10µm. (**C**) Few scattered Ki67+/tau-GFP cells (white arrowheads) were observed in Case 5, scale bar 100µm. (**D-F**) Few activated macrophages (**D**, red arrowheads, CD68+) were present near grafted sites like presented here for SN graft sites in case 5 (**D** and **E**) and case 4 (**F**); scale bars (D) 30µm, (E-F) 100µm.

Table S1: Longitudinal protocol design, repeated measures and case controls

Reference study	Protocol's Subparts	Neuroscience Disciplines	Present study cases	Total number of NHPs used
Wianny et al.,2008 *Stem Cells* [39]<br><br>Wianny et al.,2011 *Differentiation* [40]	ESCs line production and NPs derivation	Stem Cells Neurobiology	-	-
Vezoli et al., 2011 *PLoS ONE* [34]	CTR; MPTP; Spontaneous Recovery	Neurobiology of Sleep and Circadian Rhythms; Cognitive Neurobiology; Clinical Neuroscience; Neurological Disease Models	1, 2, 4	3 + monkey T
Vezoli et al., 2014 *NeuroImage* [36]	CTR; MPTP; Spontaneous Recovery; Stable Symptoms	Neurobiology of Sleep and Circadian Rhythms; Cognitive Neurobiology; Clinical Neuroscience; Neuroimaging and Systems Neuroscience; Neuroanatomy; Neurological Disease Models	1, 2, 3, 6	4
Fifel et al., 2014 *PLoS ONE* [35]	CTR; MPTP; Spontaneous Recovery; Stable Symptoms	Neurobiology of Sleep and Circadian Rhythms; Clinical Neuroscience; Neuroanatomy; Neurobiology of Disease	1-6	6 + 5 monkeys (B, C, J, R, V)
This study	CTR; MPTP; Spontaneous Recovery; Stable Symptoms; Pre- vs. Post-Graft	Neurobiology of Sleep and Circadian Rhythms; Cognitive Neurobiology; Clinical Neuroscience; Neuroimaging; Anatomy and Histology; Neurobiology of Disease; Stem Cells Neurobiology; Translational Neuroscience	1-6<br><br>3 additional control cases	6 + 3 control animals

Control procedures are detailed in the reference studies, identification of the cases used in the present study and the total number of animals used per study. Overall 15 different NHPs were involved in the longitudinal protocol design.

Table S2: Total cumulated MPTP doses per case

**CASE 1**	7.8 mg/kg (27 inj. SP – 3 months – 12 inj. HF)
**CASE 2**	4.4 mg/kg (22 inj. SP/ HF)
**CASE 3**	4.8 mg/kg (8 inj. SP – 5 months – 16 inj. SP/ HF)
**CASE 4**	4.4 mg/kg (11 inj. SP – 26 months – 11 inj. HF)
**CASE 5**	3.0 mg/kg (15 inj. SP/ HF)
**CASE 6**	2.4 mg/kg (12 inj. HF)

In cases 1, 3 and 4 MPTP intoxication was implemented in two phases with the first one consisting in chronic low-doses systemic injections (interval of at least 72-96H between injections (inj.), slowly progressive lesion (SP) that was halted in order to promote spontaneous recovery from motor symptoms – observation period in months –). In all cases, MPTP intoxication ended after a period of low-doses injections with a higher frequency (HF, interval of 24h between injections during weekdays).

Table S3. Cell survival following transplantation of NPs

	Caudate nucleus		*Putamen*		*Substantia nigra*	
	**LH**	**RH**	**LH**	**RH**	**LH**	**RH**
Case 1	3.15×10^{3}	0	4.77×10^{4}	0	3.03×10^{4}	3.12×10^{4}
**Case 2***	-	-	-	-	-	-
Case 3	0	0	0	0	0	0
Case 4	5×10^{3}	9×10^{3}	NA	NA	11×10^{4}	0
Case 5	5.67×10^{4}	0	2.52×10^{4}	4.95×10^{3}	18.4×10^{4}	$<1\times10^{2}$
Case 6	0	0	0	0	0	0

Number of tau-GFP^{+} cells estimated *post-mortem* after an average delay of 30±8 weeks post-graft. * Case 2 had per-operative brain hemorrhage during NPs transplantation. Cases 1, 4 and 5 displayed functional recovery following cell-therapy (*Graft Survival* group) whereas cases 3 and 6 did not (*No Graft Survival* group). Case 4 was not grafted with NPs in the Putamen.

Table S4: Grafted cells number, survival rates, differentiation fates and comparison to literature

Grafted cells	Grafted cells number (per site)	Grafted sites	Survival rate (%)	Graft size (mm^3)	TH$^+$	MAP2$^+$	GFAP$^+$	Graft type	Ref.
**NPs**	**0.15-1×10^6**	**CdN, Put, SN**	**7±3†**	**2.2±0.9**	**6±2%**	**16±4%**	**42±5%**	**Xeno**	**This study**
NSCs	1×10^6	CdN, SN	<10	NA	<1%	3%	NA	Xeno$^+$	[74, 120]
	NA	CdN, Put	NA	16-41	<1.1%	0%	0%	Xeno$^+$	[121]
	10×10^6	CdN, Put, SN	10%	NA	1.85%	NA	NA	Xeno$^+$	[33, 122]
	2×10^6	CdN, SN	NA	NA	NA	NA	NA	Auto	[123]
	2×10^6	CdN, Put, SN	NA	NA	NA	NA	NA	Allo	[124]
DA neurons	1.25×10^6	CdN, Put	NA	NA	NA	NA	NA	Xeno$^+$	[125]
	2.4×10^6	Put	NA	NA	<0.75%	39%	NA	Xeno$^+$	[126]
	1×10^6	CdN, Put	NA	NA	10%	NA	NA	Xeno$^+$	[127]
	2.5×10^6	Put	NA	5.4-14	<0.1%	NA	NA	Allo	[128]
	0.15-0.3×10^6	Put	1.3-2.7	NA	0.4%	NA	NA	Allo$^+$	[129]
	0.25-0.75×10^6	CdN, Put, SN	<1.5	NA	NA	63%	22%	Auto	[31]
	5×10^6	Put	NA	NA	<0.1%	NA	NA	Auto	[130, 131]
	NA	CdN, Put	NA	NA	0.4%	NA	NA	Auto	[132]
	2.4×10^6	Put	NA	39.4±21	33±24 %	NA	NA	Xeno$^+$	[133]
	0.5-7.3×10^6	CdN, Put ± SN	NA	0.63-36.5	*	NA	NA	Auto/Allo	[95]
	2×10^6	CdN, Put	6-53%	~100-150	5.2-8.1%	NA	NA	Xeno	[106]
Other	0.3-0.5×10^6	CdN or Put	NA	NA	NA	NA	NA	Xeno	[134]
	4×10^6	CdN	NA	NA	NA	NA	NA	Allo	[135]
	0.5-3×10^6	CdN, Put, SN	NA	NA	NA	NA	NA	Allo	[136]
	NA	Put	NA	NA	**	NA	NA	Allo	[137]
	0.2-0.4×10^6	CdN, Put, SN	10-50	NA	NA	NA	NA	Allo	[138]

* 0.6±0.7×10^4 (Allo) and 9.1±5.8×10^4 (Auto) TH$^+$ cells at grafted location; ** 80-100 TH$^+$ cells at grafted location;
$^+$ immunosuppressive treatment. Min-Max and Mean±SE.
†over all grafted sites

Table S5: Clinical motor, cognitive and functional impact of grafts described in Supplementary Table 4

Grafted cells	Clinical motor score	Cognitive behavior	Circadian rhythm	F-DOPA	DAT	Other, comments	Ref.
NPs	Improvement	Improvement	Partial recovery	Increase	Increase	-	This study
NSCs	Improvement	NA	NA	NA	NA	-	[74, 120]
	NA	NA	NA	NA	NA	Asymptomatic animals	[121]
	No effect	NA	NA	NA	NA	Increase in striatal DA	[33, 122]
	Improvement	NA	NA	NA	Increase	No quantification	[123]
	Improvement	NA	NA	NA	Increase	No quantification	[124]
DA neurons	NA	NA	NA	NA	NA	-	[125]
	Improvement	NA	NA	Increase	NA	-	[126]
	NA	NA	NA	NA	NA	-	[127]
	Improvement	NA	NA	Increase	NA	-	[128]
	Improvement	NA	NA	NA	NA	-	[129]
	NA	NA	NA	NA	NA	-	[31]
	NA	NA	NA	NA	NA	-	[130, 131]
	Improvement	NA	NA	NA	NA	-	[132]
	Improvement	NA	NA	NA	NA	-	[133]
	Improvement (Autograft)	Reduced depressive signs	NA	NA	NA	Increase of VMAT2 (Autograft only)	[95]
	Improvement	NA	NA	NA	NA	Increase in striatal DA (HPLC)	[106]
Other	Improvement	NA	NA	NA	NA	Change in FDG patterns	[134]
	Asymptomatic animals	NA	NA	NA	NA	Increase in striatal DA metabolite	[135]
	NA	NA	NA	NA	Increase	Graft before MPTP, increase in striatal DA, SERT	[136]
	Improvement	NA	NA	Increase	NA	-	[137]
	Improvement	NA	NA	NA	NA	-	[138]

SPECT: Single-photon emission computed tomography; FDG: fluorodeoxyglucose; VMAT2: Vesicular MonoAmine Transporter type 2; HPLC: High-Performance Liquid Chromatography.

Table S6: Antibodies list

Name	Supplier	Reference
Rabbit anti-GFAP	Dako	Z0334
Chicken anti-GFP	Life Technologies	A10262
Mouse anti-MAP2	SIGMA	M4403
Mouse anti-NESTIN	Millipore	MAB5326
Mouse Tuj1	SIGMA	T8660
Goat anti-SOX2	Santa Cruz	sc-17320
Rabbit anti-Ki67	Thermo scientific	RM-9106
Goat anti-GDNF	R&D systems	AF-212-NA*
Mouse anti-CD68	R&D systems	MAB 20401
Rabbit anti-SYNUCLEIN	ProteinTech	17785-1-AP
Rabbit anti-TYROSINE HYDROXYLASE	Chemicon	VPA151
Mouse anti- TYROSINE HYDROXYLASE	Millipore	MAB318
Rat anti-DOPAMINE TRANSPORTER (DAT)	Millipore	MAB369
Rabbit anti-SEROTONIN TRANSPORTER (SERT)	Millipore	AB9322
Goat anti-Chicken alexa 488	Life Technologies	A11039
Goat anti-Mouse alexa 647	Life Technologies	A21235
Goat anti-Rabbit alexa 555	Life Technologies	A21428
Donkey anti-Goat alexa 555	Life Technologies	A21432
Donkey anti-Rabbit alexa 647	Life Technologies	A31373
Donkey anti-Rabbit alexa 555	Life Technologies	A31572
Donkey anti-Mouse alexa 647	Life Technologies	A31571
Donkey anti-Chicken dylight 488	Jackson	703-485-155

*not shown in results

Table S7: Primers for semi-quantitative RT-PCR

Gene	Forward Primer (5′-3′)	Reverse Primer (5′-3′)
**β actin**	AAACTGGAACGGTGAAGGTG	TCAAGTTGGGGGACAAAAAGG
**AhD2**	GTTGTCAAACCAGCAGAGCA	CAAGTCGGCATCAGCTAACA
**DRD2**	GGAGGTGGTAGGTGAGTGGA	GGAGATGGTGAAGGACAGGA
**EN1**	CTAGCCAAACCGCTTACGAC	GCAGAACAGACAGACCGACA
**GAD67**	ATTCTTGAAGCCAAACAG	TAGCTTTTCCCGTCGTTG
**GFAP**	GGCAGGGCATGACTTTGTTC	TAAGAAGGGACCGCAAGAGG
**LMX1A**	CCTGCAGGAAGGTGAGAGAG	GTCGTCGCTATCCAGGTCAT
**LMX1B**	GCAGCGGCTGCATGGAGAAGATCGC	GGTTCTGAAACCAGACCTGGACAAC
**MAP2**	CAGCAAAGGGATACTTTCAC	ATGCTTTTTGTTGCTTCTTC
**MUSASHI**	CGAGCTCGACTCCAAAACAATT	TCTACACGGAATTCGGGGAACT
**NESTIN**	CGTCTTGGATCTTTGCTCCC	GGGCTCTGATCTCTGCATCT
**NGN2**	CCGAGACCTTGGAGTTGAAG	CGTTTGCAATCGTGTACCAG
**NURR1**	CTCCCAGAGGGAACTGCACTTCG	CTCTGGAGTTAAGAAATCGGAGCTG
**OCT4**	CGACCATCTGCCGCTTTGAG	CCCCCTGTCCCCCATTCCTA
**PAX2**	TGTGTCAGCAAAATCCTGGGCAGGT	TGCTGAACTTTGGTCCGGATGAT
**PAX6**	CATGCAGAACAGTCACAGCGG	CCCATCTGTTGCTTTTCGCTA
**SOX2**	CCCCCGGCGGCAATAGCA	TCGGCGCCGGGGAGATACAT
**TH**	ATGGCTGAGCCCCGCCAGGAG	TCACAAACCCTGCTTGGCC
**c-RET**	CGACCTCATCTCATTTGCC	AATCTTCATCTTCCGCCCC

Table S8: Mixed-effects stats of Fig.3A for the *Graft Survival* group

		PMRS	ORDT success	ORDT errors	LD ratio
**Fixed effects:**		β, SE, 95% CI, t-value	β, SE, 95% CI, t-value	β, SE, 95% CI, t-value	β, SE, 95% CI, t-value
**MPTP**	**Q1**	0.58, 2.14, -2.89–4.08, 0.27	-0.12, 0.15, -0.34–0 .11, -0.79	0.20, 0.13, -0.02–0.39, 1.46	-0.05, 0.09, -0.20–0.09, -0.55
	**Q2**	2.37, 2.14, -1.10–5.88, 1.11	-0.25, 0.13, -(0.45–0.04), -1.84	0.23, 0.12, 0.04–0.41, 1.90	-0.18, 0.09, -(0.32–0.03), -2.03
	**Q3**	4.83, 2.15, 1.32–8.33, 2.25	-0.43, 0.14, -(0.64–0.20), -3.02	0.25, 0.13, 0.04–0.43, 1.97	-0.23, 0.09, -(0.38–0.08), -2.62
	**Q4**	5.55, 2.14, 2.05–9.04, 2.59	-0.33, 0.14, -(0.53–0.10), -2.40	0.26, 0.12, 0.04–0.43, 2.10	-0.27, 0.09, -(0.42–0.13), -3.09
	**Q5**	9.96, 2.14, 6.45–13.43, 4.64	-0.33, 0.14, -(0.55–0.09), -2.27	0.27, 0.13, 0.05–0.47, 2.02	-0.32, 0.09, -(0.46–0.13), -3.67
**Stage II**	**Q1**	13.04, 2.13, 9.56–16.48, 6.12	-0.39, 0.14, -(0.60–0.17), -2.84	0.37, 0.12, 0.15–0.54, 2.92	-0.31, 0.09, -(0.44–0.16), -3.58
	**Q2**	11.03, 2.13, 7.54–14.47, 5.17	-0.65, 0.15, -(0.87–0.43), -4.39	0.70, 0.13, 0.51–0.89, 5.34	-0.32, 0.08, -(0.46–0.18), -3.78
	**Q3**	11.17, 2.13, 7.69–14.61, 5.24	-0.59, 0.15, -(0.81–0.34), -3.88	0.47, 0.14, 0.24–0.66, 3.41	-0.40, 0.09, -(0.53–0.25), -4.64
	**Q4**	12.20, 2.13, 8.71–15.65, 5.71	-0.71, 0.15, -(0.96–0.48), -4.60	0.40, 0.14, 0.18–0.60, 2.90	-0.37, 0.09, -(0.51–0.23), -4.32
	**Q5**	10.80, 2.14, 7.29–14.27, 5.04	-0.50, 0.14, -(0.71–0.28), -3.61	0.50, 0.12, 0.30–0.68, 3.98	-0.29, 0.09, -(0.43–0.14), -3.29
**Post-graft**	**Q1**	6.40, 2.12, 2.96–9.83, 3.02	-0.38, 0.13, -(0.57–0.19), -2.94	0.24, 0.11, 0.07–0.40, 2.12	-0.34, 0.08, -(0.47–0.20), -4.03
	**Q2**	2.67, 2.12, -0.77–6.10, 1.26	-0.15, 0.13, -0.34–0.05, -1.14	0.18, 0.11, 0.01–0.34, 1.61	-0.22, 0.08, -(0.35–0.08), -2.60
	**Q3**	2.68, 2.12, -0.75–6.11, 1.26	-0.24, 0.14, -(0.45–0.02), -1.63	0.16, 0.13, -0.03–0.34, 1.28	-0.24, 0.08, -(0.38–0.10), -2.80
	**Q4**	2.29, 2.12, -1.14–5.73, 1.08	-0.13, 0.15, -0.35–0.09, -0.90	0.08, 0.13, -0.12–0.26, 0.59	-0.12, 0.08, -0.26–0.01, -1.51
	**Q5**	2.66, 2.12, -0.78–6.10, 1.25	-0.03, 0.13, -0.22–0.17, -0.26	>0.01, 0.12, -0.18–0.17, 0.03	-0.11, 0.08, -0.25–0.02, -1.36
**Random effects:**					
	Case	(n=3, SD=1.40)	(n=3, SD=0.27)	(n=3, SD = 0.27)	(n=3, SD=0.06)
	Case | condition	(n=48, SD=2.58)	(n=42, SD=0.15)	(n=42, SD = 0.13)	(n=48, SD=0.10)
**Observations**		870	283	283	825
**R^2m / R^2c**		0.601 / 0.911	0.238 / 0.855	0.210 / 0.831	0.299 / 0.607

Linear mixed-effects: fixed effects *i.e.*, first level contrast (estimate – β, standard error – SE, 95% confidence intervals determined by profiling – CI, t-value); random effects (groups, standard deviation – SD); Number of Observations, marginal and conditional R^2. Significant *within-subject control* difference (first level contrast) based on exclusion of zero in the 95% CI calculated by a profiling method and highlighted in red (see Methods). PMRS: Parkinsonian Monkey Rating Scale – Clinical motor score; ORDT: object retrieval detour task – Cognitive performances; LD ratio: Light/Dark ratio – Chronobiological rhythms.

Table S9: Mixed-effect stats of Figure 3A for the *No Graft Survival* group

		PMRS	ORDT success	ORDT errors	LD ratio
**Fixed effects:**		β, SE, 95% CI, t-value	β, SE, 95% CI, t-value	β, SE, 95% CI, t-value	β, SE, 95% CI, t-value
**MPTP**	**Q1**	0.24, 2.57, -3.33–3.83, 0.09	-0.29, 0.10, -(0.46–0.11), -2.75	0.17, 0.19, 0.01–0.30, 0.92	-0.05, 0.19, -0.30–0.18, -0.28
	**Q2**	0.31, 2.58, -3.27–3.91, 0.12	-0.31, 0.10, -(0.47–0.14), -3.12	0.16, 0.19, -0.04–0.23, 0.83	0.06, 0.19, -0.20–0.29, -0.30
	**Q3**	0.84, 2.57, -2.72–4.42, 0.32	-0.27, 0.10, -(0.43–0.07), -2.49	0.003, 0.19, -0.03–0.26, 0.02	-0.07, 0.19, -0.32–0.16, -0.40
	**Q4**	3.88, 2.57, 0.31–7.45, 1.51	-0.38, 0.10, -(0.51–0.18), -3.76	0.31, 0.19, 0.18–0.44, 1.59	-0.11, 0.19, -0.36–0.12, -0.63
	**Q5**	6.72, 2.60, 3.26–1.57, 2.58	-0.76, 0.21, -(1.12–0.35), -3.50	0.84, 0.27, 0.56–1.19, 3.13	0.03, 0.20, -0.29–0.23, 0.18
**Stage II**	**Q1**	11.1, 2.56, 7.69–14.75, 4.38	-0.69, 0.13, -(0.96–0.49), -5.43	0.53, 0.23, 0.30–0.67, 2.31	-0.10, 0.18, -0.33–0.13, -0.56
	**Q2**	11.9, 2.57, 8.41–15.52, 4.64	-0.81, 0.22, -(1.25–0.44), -3.69	0.28, 0.27, -0.08–0.55, 1.06	-0.27, 0.18, -(0.50–0.03), -1.44
	**Q3**	11.7, 2.57, 8.13–15.23, 4.55	-0.81, 0.22, -(1.25–0.44, -3.69	0.70, 0.27, 0.33–0.97, 2.61	-0.26, 0.18, -(0.49–0.03), -1.41
	**Q4**	12.5, 3.14, 8.10–16.9, 3.98	NA	NA	-0.31, 0.18, -(0.59–0.02), -1.38
	**Q5**	11.0, 2.56, 7.45–14.52, 4.29	NA	NA	-0.21, 0.23, -0.45–0.02, -1.16
**Post-graft**	**Q1**	9.33, 2.55, 5.81–12.84, 3.65	-0.69, 0.15, -(1.00–0.46), -4.73	0.64, 0.24, 0.39–0.81, 2.73	-0.19, 0.18, -0.41–0.04, -1.02
	**Q2**	8.13, 2.55, 4.61–11.64, 3.18	-0.39, 0.11, -(0.63–0.23), -3.58	0.44, 0.22, 0.24–0.54, 1.94	-0.11, 0.18, -0.34–0.12, -0.60
	**Q3**	7.98, 2.55, 4.47–11.50, 3.13	-0.41, 0.11, -(0.65–0.24), -3.72	0.48, 0.22, 0.28–0.59, 2.15	-0.05, 0.18, -0.27–0.18, -0.25
	**Q4**	7.98, 2.55, 4.46–11.50, 3.13	-0.63, 0.12, -(0.89–0.44), -5.15	0.54, 0.23, 0.32–0.66, 2.36	-0.08, 0.18, -0.31–0.15, -0.46
	**Q5**	8.46, 2.55, 4.94–11.98, 3.31	-0.63, 0.12, -(0.89–0.44), -5.15	0.56 0.23, 0.34–0.68, 2.44	-0.16, 0.23, -0.45–0.12, -0.69
**Random effects:**					
	Case	(n=2, SD=0.441)	(n=2, SD=0.116)	(n=2, SD=0.063)	(n=2, SD=0.145)
	Case | condition	(n=31, SD=2.539)	(n=20, SD=0.042)	(n=20, SD=0.173)	(n=30, SD=0.181)
**Observations**		n=729	n=88	n=88	n=659
**R^2m / R^2c**		0.599 / 0.899	0.468 / 0.616	0.476 / 0.787	0.091 / 0.701

Within-subject control difference (first level contrast). Same conventions as in Table S8.

Table S10: Mixed-effect stats of Figure 4A for the *Spontaneous Recovery* group

		PMRS – Spontaneous Recovery	PMRS – Graft-induced Recovery
**Fixed effects:**		β, SE, 95% CI, t-value	β, SE, 95% CI, t-value
**Weeks**	**1**	8.73, 1.09, 6.95–10.5, 8.02	9.92, 1.31, 7.73–12.1, 7.59
	**2**	7.23, 1.08, 5.45–9.01, 6.66	7.59, 1.31, 5.40–9.78, 5.78
	**3**	4.02, 1.09, 2.24–5.80, 3.70	6.31, 1.31, 4.12–8.50, 4.83
	**4**	2.27, 1.09, 0.49–4.05, 2.09	4.72, 1.31, 2.53–6.91, 3.61
	**5**	1.36, 1.09, -0.42–3.14, 1.25	3.94, 1.31, 1.76–6.13, 3.02
	**6**	0.86, 1.09, -0.92–2.65, 0.79	2.96, 1.31, 0.78–5.15, 2.27
	**7**	0.61, 1.20, -1.35–2.56, 0.51	3.58, 1.31, 1.39–5.77, 2.74
	**8**	0.31, 1.09, -1.47–2.10, 0.29	2.93, 1.31, 0.74–5.12, 2.24
	**9**	0.34, 1.09, -1.45–2.11, 0.31	2.40, 1.31, 0.21–4.59, 1.84
	**10**	0.33, 1.09, -1.45–2.12, 0.31	2.31, 1.31, 0.12–4.50, 1.77
	**11**	0, 1.20, -1.96–1.96, 0	2.46, 1.31, 0.27–4.65, 1.88
	**12**	0.40, 1.20, -1.57–2.36, 0.33	2.44, 1.31, 0.26–4.63, 1.87
	**13**	0, 1.20, -1.96–1.96, 0	2.83, 1.31, 0.64–5.02, 2.17
**Random effects:**			
	Case	(n=3, SD=0.0001)	(n=3, SD=0.0002)
	Case | condition	(n=38, SD=1.223)	(n=42, SD=0.809)
**Observations**		n=229	n=274
**R^2m / R^2c**		0.802 / 0.952	0.650 / 0.919

Within-subject control difference (first level contrast). Spontaneous recovery group: Cases 1, 3 and 4; Graft-induced Recovery group: Cases 1, 4 and 5. Same conventions as in Table S8.

Table S11: Mixed-effect stats of Figure 4B for the *Spontaneous Recovery* group

		ORDT – Spontaneous Recovery	
		success	errors
Fixed effects:		β, SE, 95% CI, t-value	β, SE, 95% CI, t-value
Post-MPTP	Q1	-0.49, 0.08, -(0.63–0.34), -5.84	0.46, 0.08, 0.32–0.59, 5.55
	Q2	-0.50, 0.08, -(0.65–0.36), -5.99	0.49, 0.08, 0.35–0.63, 5.96
	Q3	-0.59, 0.08, -(0.74–0.45), -7.03	0.49, 0.08, 0.35–0.63, 5.84
	Q4	-0.45, 0.09, -(0.60–0.30), -5.11	0.39, 0.09, 0.25–0.54, 4.52
	Q5	-0.41, 0.10, -(0.64–0.30), -4.72	0.38, 0.10, 0.21–0.55, 3.88
**Random effects:**			
	Case	(n=3, SD=0.156)	(n=3, SD=0.167)
	Case | condition	(n=17, SD=0.084)	(n=17, SD=0.081)
**Observations**		n=131	n=131
**R^2m / R^2c**		0.370 / 0.721	0.301 / 0.699

Within-subject control difference (first level contrast). Spontaneous recovery group: Cases 1, 3 and 4; Graft-induced Recovery group: Cases 1, 4 and 5. Same conventions as in Table S8.

Supporting Information References

[100] Benazzouz, A., T. Boraud, P. Dubedat, A. Boireau, J.M. Stutzmann, and C. Gross, *Eur J Pharmacol*, *1995*. 284(3): p. 299-307.

[101] Hantraye, P., M. Varastet, M. Peschanski, D. Riche, P. Cesaro, J.C. Willer, and M. Maziere, *Neuroscience*, **1993**. 53(1): p. 169-178.

[102] Emborg, M.E., J.W. Tetrud, J. Moirano, W.W. McLaughlin, and K.S. Bankiewicz, *Front Biosci*, **2003**. 8: p. a148-154.

[103] Bergman, H., A. Raz, A. Feingold, A. Nini, I. Nelken, D. Hansel, H. Ben-Pazi, and A. Reches, *Mov Disord*, **1998**. 13 Suppl 3: p. 29-34.

[104] Choudhury, G.R. and M.M. Daadi, *PLoS One*, **2018**. 13(8): p. e0202770.

[105] Araki, R., M. Uda, Y. Hoki, M. Sunayama, M. Nakamura, S. Ando, M. Sugiura, H. Ideno, A. Shimada, A. Nifuji, and M. Abe, *Nature*, **2013**. 494(7435): p. 100-104.

[106] Wang, Y.K., W.W. Zhu, M.H. Wu, Y.H. Wu, Z.X. Liu, L.M. Liang, C. Sheng, J. Hao, L. Wang, W. Li, Q. Zhou, and B.Y. Hu, *Stem Cell Reports*, **2018**. 11(1): p. 171-182.

[107] Doxiadis, G.G., A.J. Rouweler, N.G. de Groot, A. Louwerse, N. Otting, E.J. Verschoor, and R.E. Bontrop, *Immunogenetics*, **2006**. 58(4): p. 259-268.

[108] Bunlungsup, S., S. Kanthaswamy, R.F. Oldt, D.G. Smith, P. Houghton, Y. Hamada, and S. Malaivijitnond, *Am J Primatol*, **2017**. 79(12).

[109] Morizane, A., T. Kikuchi, T. Hayashi, H. Mizuma, S. Takara, H. Doi, A. Mawatari, M.F. Glasser, T. Shiina, H. Ishigaki, Y. Itoh, K. Okita, E. Yamasaki, D. Doi, H. Onoe, K. Ogasawara, S. Yamanaka, and J. Takahashi, *Nat Commun*, **2017**. 8(1): p. 385.

[110] Diamond, A., *Philos Trans R Soc Lond B Biol Sci*, **1996**. 351(1346): p. 1483-1493; discussion 1494.

[111] Schneider, J.S., M. Van Velson, F. Menzaghi, and G.K. Lloyd, *Ann Neurol*, **1998**. 43(3): p. 311-317.

[112] Taylor, J.R., J.D. Elsworth, R.H. Roth, T.J. Collier, J.R. Sladek, Jr., and D.E. Redmond, Jr., *Prog Brain Res*, **1990**. 82: p. 543-559.

[113] Taylor, J.R., J.D. Elsworth, R.H. Roth, J.R. Sladek, Jr., and D.E. Redmond, Jr., *Brain*, **1990**. 113 (Pt 3): p. 617-637.

[114] Dkhissi-Benyahya, O., C. Gronfier, W. De Vanssay, F. Flamant, and H.M. Cooper, *Neuron*, **2007**. 53(5): p. 677-687.

[115] Emond, P., L. Garreau, S. Chalon, M. Boazi, M. Caillet, J. Bricard, Y. Frangin, L. Mauclaire, J.C. Besnard, and D. Guilloteau, *J Med Chem*, **1997**. 40(9): p. 1366-1372.

[116] Poyot, T., F. Conde, M.C. Gregoire, V. Frouin, C. Coulon, C. Fuseau, F. Hinnen, F. Dolle, P. Hantraye, and M. Bottlaender, *J Cereb Blood Flow Metab*, **2001**. 21(7): p. 782-792.

[117] Shih, M.C., M.Q. Hoexter, L.A. Andrade, and R.A. Bressan, *Sao Paulo Med J*, **2006**. 124(3): p. 168-175.

[118] Paxinos, G., A.W. Toga, and X.F. Huang, The rhesus monkey brain in stereotaxic coordinates / George Paxinos, Xu-Feng Huang, Arthur Toga, *ed. A.W. Toga and X.F. Huang* **2000**, San Diego, Calif: Academic Press.

[119] Dzahini, K., C. Dentresangle, M. Le Cavorsin, A. Bertrand, I. Detraz, M. Savasta, and V. Leviel, *J Neurochem*, **2010**. 113(6): p. 1459-1470.

[120] Bjugstad, K.B., Y.D. Teng, D.E. Redmond, Jr., J.D. Elsworth, R.H. Roth, S.K. Cornelius, E.Y. Snyder, and J.R. Sladek, Jr., *Exp Neurol*, **2008**. 211(2): p. 362-369.

[121] Wakeman, D.R., S. Weiss, J.R. Sladek, J.D. Elsworth, B. Bauereis, C. Leranth, P.J. Hurley, R.H. Roth, and D.E. Redmond, *Cell Transplant*, **2014**. 23(8): p. 981-994.

[122] Gonzalez, R., I. Garitaonandia, A. Crain, M. Poustovoitov, T. Abramihina, A. Noskov, C. Jiang, R. Morey, L.C. Laurent, J.D. Elsworth, E.Y. Snyder, D.E. Redmond, Jr., and R. Semechkin, *Cell Transplant*, **2015**. 24(4): p. 681-690.

[123] Xu, Q., X. Jiang, Y. Ke, S. Zhang, R. Xu, and Y. Zeng, *Cytotherapy*, **2010**. 12(2): p. 226-237.

[124] Zhou, Y., M. Sun, H. Li, M. Yan, Z. He, W. Wang, and S. Lu, *Cytotherapy*, **2013**. 15(4): p. 467-480.

[125] Kriks, S., J.W. Shim, J. Piao, Y.M. Ganat, D.R. Wakeman, Z. Xie, L. Carrillo-Reid, G. Auyeung, C. Antonacci, A. Buch, L. Yang, M.F. Beal, D.J. Surmeier, J.H. Kordower, V. Tabar, and L. Studer, *Nature*, **2011**. 480(7378): p. 547-551.

[126] Doi, D., A. Morizane, T. Kikuchi, H. Onoe, T. Hayashi, T. Kawasaki, M. Motono, Y. Sasai, H. Saiki, M. Gomi, T. Yoshikawa, H. Hayashi, M. Shinoyama, M.M. Refaat, H. Suemori, S. Miyamoto, and J. Takahashi, *Stem Cells*, **2012**. 30(5): p. 935-945.

[127] Daadi, M.M., B.A. Grueter, R.C. Malenka, D.E. Redmond, Jr., and G.K. Steinberg, *PLoS ONE*, **2012**. 7(7): p. e41120.

[128] Sanchez-Pernaute, R., L. Studer, D. Ferrari, A. Perrier, H. Lee, A. Vinuela, and O. Isacson, *Stem Cells*, **2005**. 23(7): p. 914-922.

[129] Takagi, Y., J. Takahashi, H. Saiki, A. Morizane, T. Hayashi, Y. Kishi, H. Fukuda, Y. Okamoto, M. Koyanagi, M. Ideguchi, H. Hayashi, T. Imazato, H. Kawasaki, H. Suemori, S. Omachi, H. Iida, N. Itoh, N. Nakatsuji, Y. Sasai, and N. Hashimoto, *J Clin Invest*, **2005**. 115(1): p. 102-109.

[130] Sundberg, M., H. Bogetofte, T. Lawson, J. Jansson, G. Smith, A. Astradsson, M. Moore, T. Osborn, O. Cooper, R. Spealman, P. Hallett, and O. Isacson, *Stem Cells*, **2013**. 31(8): p. 1548-1562.

[131] Hallett, P.J., M. Deleidi, A. Astradsson, G.A. Smith, O. Cooper, T.M. Osborn, M. Sundberg, M.A. Moore, E. Perez-Torres, A.L. Brownell, J.M. Schumacher, R.D. Spealman, and O. Isacson, *Cell Stem Cell*, **2015**. 16(3): p. 269-274.

[132] Wang, S., C. Zou, L. Fu, B. Wang, J. An, G. Song, J. Wu, X. Tang, M. Li, J. Zhang, F. Yue, C. Zheng, P. Chan, Y.A. Zhang, and Z. Chen, *Cell Discov*, **2015**. 1: p. 15012.

[133] Kikuchi, T., A. Morizane, D. Doi, H. Onoe, T. Hayashi, T. Kawasaki, H. Saiki, S. Miyamoto, and J. Takahashi, *J Parkinsons Dis*, **2011**. 1(4): p. 395-412.

[134] Peng, S., Y. Ma, J. Flores, M. Cornfeldt, B. Mitrovic, D. Eidelberg, and D.J. Doudet, *J Nucl Med*, **2016**. 57(10): p. 1591-1598.

[135] Wolff, E.F., L. Mutlu, E.E. Massasa, J.D. Elsworth, D. Eugene Redmond, Jr., and H.S. Taylor, *J Cell Mol Med*, **2015**. 19(1): p. 249-256.

[136] Ren, Z., J. Wang, S. Wang, C. Zou, X. Li, Y. Guan, Z. Chen, and Y.A. Zhang, *Sci Rep*, **2013**. 3: p. 2786.

[137] Luquin, M.R., M. Manrique, J. Guillen, J. Arbizu, C. Ordonez, and I. Marcilla, *Brain Res*, **2011**. 1375: p. 120-127.

[138] Bloch, J., J.F. Brunet, C.R. McEntire, and D.E. Redmond, *J Comp Neurol*, **2014**. 522(12): p. 2729-2740.
